# The combination of Tanshinone IIA and Astragaloside IV attenuates myocardial ischemia–reperfusion injury by inhibiting the STING pathway

**DOI:** 10.1186/s13020-024-00908-y

**Published:** 2024-02-28

**Authors:** Pan Zhai, Qianyun Chen, Xunxun Wang, Xiaohu Ouyang, Mengling Yang, Yalan Dong, Junyi Li, Yiming Li, Shanshan Luo, Yue Liu, Xiang Cheng, Rui Zhu, Desheng Hu

**Affiliations:** 1 Department of Integrated Traditional Chinese and Western Medicine, Union Hospital, Tongji Medical College, Huazhong University of Science and Technology, Wuhan, 430022 China; 2 Department of Critical Care Medicine, Zhongnan Hospital of Wuhan University, Wuhan, 430071 China; 3 Institute of Hematology, Union Hospital, Tongji Medical College, Huazhong University of Science and Technology, Wuhan, 430022 China; 4 Cardiovascular Disease Center, Xiyuan Hospital of China Academy of Chinese Medical Sciences, Beijing, 100091 China; 5 Department of Cardiology, Union Hospital, Tongji Medical College, Huazhong University of Science and Technology, Wuhan, 430022 China; 6 Hubei Key Laboratory of Biological Targeted Therapy, Union Hospital, Tongji Medical College, Huazhong University of Science and Technology, Wuhan, 430022 China

**Keywords:** Tanshinone IIA, Astragaloside IV, Myocardial ischemia reperfusion injury, STING pathway, Apoptosis, Oxidative stress

## Abstract

**Background:**

Astragaloside IV (As-IV) and Tanshinone IIA (Ta-IIA) are the main ingredients of traditional Chinese medicinal *Astragalus membranaceus* (Fisch.) Bunge and *Salvia miltiorrhiza* Bunge, respectively, both of which have been employed in the treatment of cardiovascular diseases. Nevertheless, the efficacy of the combination (Co) of Ta-IIA and As-IV for cardiovascular diseases remain unclear and warrant further investigation. This study aimed to investigate the efficacy and the underlying molecular mechanism of Co in treating myocardial ischemia–reperfusion injury (MIRI).

**Methods:**

In order to assess the efficacy of Co, an in vivo MIRI mouse model was created by temporarily blocking the coronary arteries for 30 min and then releasing the blockage. Parameters such as blood myocardial enzymes, infarct size, and ventricular function were measured. Additionally, in vitro experiments were conducted using HL1 cells in both hypoxia-reoxygenation model and oxidative stress models. The apoptosis rate, expression levels of apoptosis-related proteins, oxidative stress indexes, and release of inflammatory factors were detected. Furthermore, molecular docking was applied to examine the binding properties of Ta-IIA and As-IV to STING, and western blotting was performed to analyze protein expression of the STING pathway. Additionally, the protective effect of Ta-IIA, As-IV and Co via inhibiting STING was further confirmed in models of knockdown STING by siRNA and adding STING agonist.

**Results:**

Both in vitro and in vivo data demonstrated that, compared to Ta-IIA or As-IV alone, the Co exhibited superior efficacy in reducing the area of myocardial infarction, lowering myocardial enzyme levels, and promoting the recovery of myocardial contractility. Furthermore, the Co showed more potent anti-apoptosis, antioxidant, and anti-inflammation effects. Additionally, the Co enhanced the inhibitory effects of Ta-IIA and As-IV on STING phosphorylation and the activation of STING signaling pathway. However, the administration of a STING agonist attenuated the protective effects of the Co, Ta-IIA, and As-IV by compromising their anti-apoptotic, antioxidant, and anti-inflammatory effects in MIRI.

**Conclusion:**

Compared to the individual administration of Ta-IIA or As-IV, the combined treatment demonstrated more potent ability in inhibiting apoptosis, oxidative stress, inflammation, and the STING signaling pathway in the context of MIRI, indicating a more powerful protective effect against MIRI.

**Graphical Abstract:**

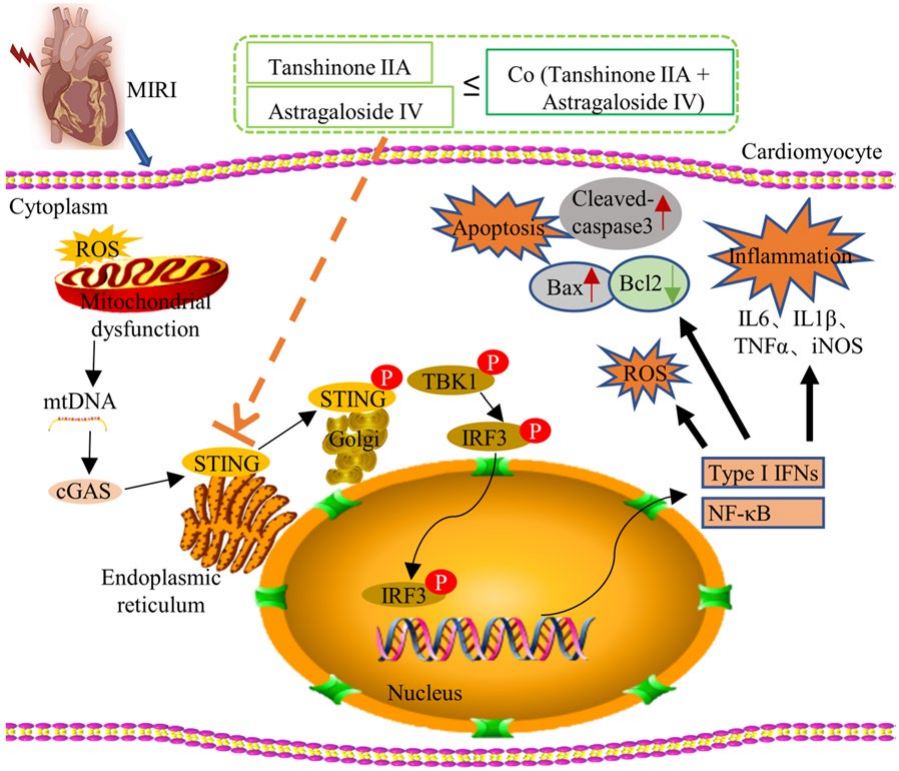

**Supplementary Information:**

The online version contains supplementary material available at 10.1186/s13020-024-00908-y.

## Introduction

Acute myocardial infarction (AMI) is a prevalent and severe form of heart disease, and vascular recanalization therapy is widely employed as a primary treatment for AMI [1]. However, the occurrence of myocardial ischemia–reperfusion injury (MIRI) significantly hampers the success rate of treating AMI and may even exacerbate the deterioration of myocardial function, posing a grave threat to patient's health and life [2]. However, there is no recommended treatment specifically for MIRI [3, 4].

MIRI usually leads to myocardial mitochondrial damage and dysfunction. In the presence of mitochondrial dysfunction, nuclear DNA damage, or bacterial invasion, the resulting DNA fragments are recognized by the cyclic GMP-AMP synthase (cGAS) enzyme in the cytoplasm [5]. This leads to the activation of cGAS, which generates a molecule called 2′3’-cyclic GMP-AMP (cGAMP) as a second messenger. Subsequently, cGAMP binds to the stimulator of interferon genes (STING) protein in the endoplasmic reticulum, triggering the activation of STING [6]. STING activates TANK-binding kinase 1 (TBK1), which proceeds to phosphorylate interferon regulatory factor 3 (IRF3) [7]. Upon translocation from the cytoplasm to the nucleus, IRF 3 interacts with other transcription factors, leading to the upregulation of interferon and other genes associated with immune responses, thereby amplifying the cellular immune response [8]. The STING signaling pathway plays a critical role in intracellular immune responses [9, 10].

It reported that the STING signaling pathway in cardiomyocytes becomes excessively activated in cardiovascular diseases [11, 12]. This abnormal activation may result from mitochondrial dysfunction caused by oxidative stress, which leads to the release of mitochondrial DNA (mtDNA), or result from nuclear DNA damage that allows DNA fragments to enter the cytoplasm [13, 14]. In either case, this further exacerbates the damage to the cardiomyocytes. Thus, targeting the STING signaling pathway could be a potential strategy for treating MIRI [13]. Several studies have demonstrated that inhibiting the activity of cGAS or STING can reduce the inflammatory response and damage caused by ischemia–reperfusion injury [15, 16].

Traditional Chinese medicine (TCM) has accumulated extensive experiences in the treatment of cardiovascular diseases, commonly using the principle of "Replenishing Qi and activating blood" in TCM [17]. *Astragalus membranaceus* (Fisch.) Bunge ("Huangqi" in Chinese), representing the "Replenishing Qi" category, and *Salvia miltiorrhiza* Bunge("Danshen" in Chinese) representing the "activating blood" category, are well-known traditional Chinese medicines [18–20]. The combined use of *Astragalus membranaceus* (Fisch.) Bunge and *Salvia miltiorrhiza* Bunge, along with other traditional Chinese medicals, can have a synergistic effect in treating cardiovascular diseases [21]. Tanshinone IIA (Ta-IIA), the main active ingredient in *Salvia miltiorrhiza* Bunge [19, 22, 23], and Astragaloside IV (As-IV), the main active ingredient in *Astragalus membranaceus* (Fisch.) Bunge [24, 25], have antioxidant, anti-inflammatory properties, and are widely used in the treatment of cardiovascular diseases in clinic in China [26, 27]. In addition, Ta-IIA can inhibit platelet aggregation and prevent blood clots formation [19], As-IV is capable of regulating immune function by modulating the polarization of macrophages [28]. Therefore, Ta-IIA and As-IV play pivotal roles in the treatment of cardiovascular diseases including MIRI, potentially with a synergistic effect. However, further research and clinical validation are necessary to identify for the specific treatment regimens and mechanisms.

The objective of this study is to compare the effects of As-IV, Ta-IIA, and the combination of As-IV and Ta-IIA (Co) in the treatment of MIRI, further elucidate the mechanism underlying the effects of As-IV, Ta-IIA and Co in treating MIRI, and investigate the involvement of the STING signaling pathway in MIRI.

## Materials and methods

### Materials

Tanshinone IIA (CAS NO:568-72-9) and Astragaloside IV (CAS NO: 84687-43-4) were purchased from Chengdu Herbpurify Co., Ltd (Chengdu, China). The diABZI STING agonist (Compound 3)(CAS NO: 2138498-18-5) was acquired from Selleck Chemicals (Houston, USA). Other important materials, including reagents, compounds, reagent kits, etc., can be found in Additional file 2: Tables S1 and S2.

### Animal management and treatment

In this study, 8-9 week-old male C57BL/6 mice were obtained from Vital River (Beijing Vital River Laboratory Animal Technology Co., Ltd., China). The mice underwent acclimated and were housed in a specific-pathogen-free breeding room located in the animal center of Tongji Medical College, Huazhong University of Science and Technology (HUST). All animal experiments strictly adhered to the guidelines established by the Animal Research Institute Committee, which were approved by the Institutional Animal Care and Use Committee (IACUC) of HUST.

The mice were randomly divided into different groups for the experiment, including the sham group (sham), ischemia/reperfusion (IR) group, IR + As-IV group, IR + Ta-IIA group, and IR + Co group, with each group consisting of six mice. The dosages of As-IV and Ta-IIA were determined based on previous studies [24, 26], The sham and IR groups were given PBS. In the drug treatment groups, intraperitoneal injections of As-IV (15 mg/kg/day), Ta-IIA (10 mg/kg/day) and the combination therapy were initiated 7 days prior to the IR surgery and continued until 7 days after the surgery. For the combination therapy group, we established three different dose groups, namely low-dose group (Ta-IIA 5 mg/kg/day + As-IV 10 mg/kg/day), medium-dose group (Ta-IIA 10 mg/kg/day + As-IV 15 mg/kg/day) and high-dose group (Ta-IIA 15 mg/kg/day + As-IV 20 mg/kg/day), and selected the most appropriate dose group for further study.

In experiments involving the administration of diABZI compound 3, a STING agonist, the following groups were established: sham group, IR group, IR + As-IV group, IR + Ta-IIA group, IR + Co group, IR + As-IV + diABZI group, IR + Ta-IIA + diABZI group, and IR + Co + diABZI group. DiABZI was administered via the tail vein at a dosage of 3 mg/kg/day, starting immediately after reperfusion surgery and given every other day for a total of 3 doses.

### Establishment and administration of MIRI

All the procedures were conducted in accordance with the experimental model of myocardial ischemia and infarction guidelines. The mice were administered pentobarbital (1%, 40 mg/kg) via intraperitoneal injection and positioned supine. The left chest fur of each mouse was shaved using a specialized razor, and the surgical site was sterilized with iodine and a solution containing 75% ethanol. Endotracheal intubation was performed, and a ventilator was applied for air supply. Subsequently, the subcutaneous pectoralis muscle was gently separated, and a 3–4 intercostal space incision was made along the left border of the sternum for each mouse. Under microscopic observation, the left atrial appendage (LAD) was ligated. Myocardial ischemia was induced for a duration of 30 min, following which the slipknot was released, and the thoracic cavity was meticulously closed in layers. The mice were subsequently extubated and allowed to recover naturally.

### Detection of serum creatine kinase (CK), creatine kinase isoenzyme MB (CKMB), and lactate dehydrogenase (LDH)

One day after the IR surgery, the mice were anesthetized, and blood samples were collected from the orbital venous plexus. Serum levels of CK, CKMB, and LDH were determined with a mouse CK assay kit, a CKMB isoenzyme assay kit, and an LDH Cytotoxicity Assay Kit (Nanjing Jiancheng Bioengineering Institute. Nanjing, China), respectively. The optical density(OD) of each sample was measured with a microplate reader (Agilent Technologies, California, USA). The CK assay was read at a wavelength of 660 nm, the CKMB assay at 340 nm, and the LDH assay at 490 nm.

### Myocardial infarct area detection

Following a reperfusion period of 3 days, the mice were compassionately sacrificed and secured in a supine position on the surgical table while under anesthesia. Subsequently, an injection of 1 mL Evans blue (3%) was administrated through both the inferior vena cava and the right atrium. After a brief waiting period of 2 min, the hearts were extracted and subjected to freezing. The frozen hearts were dissected into 2.5 mm sections along their longitudinal axis. These heart sections were then immersed in a solution of 2% triphenyltetrazolium chloride (TTC) and incubated for 20 min at a temperature of 37 ℃ in darkness. Following this, the heart sections were transferred to a solution of 4% paraformaldehyde and stored overnight in darkness at a temperature of 4 ℃. In the resultant stained images, the color blue denoted normal myocardial tissue, red indicated the area of risk, and white represented the infarcted region. The areas were precisely quantified using Image Pro Plus 6.0 software (Media Cybernetics, Inc. USA), and the percentage of the infarcted area was expressed as the percentage of the white area of each section of the total heart area.

### Echocardiography

To evaluate the cardiac functions of 8–9-week-old mice weighing 23–25 g, M-mode echocardiography was performed using the Vevo2100 Imaging System from VisualSonics (Toronto, Canada). The system was equipped with a 10-MH2 phased-array transducer. A medical ultrasonic couplant from Tianjin Yajie Medical Material Co., Ltd.(Tianjin, China) was then applied to the mice. Two-dimensional targeted M-mode traces were recorded from the parasternal short-axis view at the mid-papillary muscle level and from the parasternal long-axis view just below the papillary muscle. A minimum of six consecutive cardiac cycles were captured and analyzed to determine the left ventricular systolic diameter (LVIDs), left ventricular diastolic diameter (LVIDd), left ventricular end diastolic volume (LVEDV), and left ventricular end systolic volume (LVESV). Finally, the ejection fraction (EF) was calculated using the formula EF = (LVEDV × LVESV)/LVEDV × 100% and the fractional shortening (FS) was determined by (LVIDd‒LVIDs)/LVIDd × 100%. The outcomes were based on three consecutive beat measurements.

### Hematoxylin and eosin (HE) staining

The mouse hearts were immersed in ice-cold phosphate-buffered saline (PBS), fixed in 4% paraformaldehyde, subjected to gradient dehydration and paraffin embedding, and then sectioned into 5 μm sections. These sections were then deparaffinized, stained with hematoxylin and eosin, dehydrated, permeabilized, sealed, and photographed for subsequent analysis.

### Extraction of myocardial tissue proteins and western blot analysis

The hearts of mice (n = 6 per group) were homogenized using RIPA lysis buffer supplemented with phenylmethylsulfonyl fluoride (PMSF) and phosphatase inhibitors. The resulting supernatant was collected after centrifugation. Protein quantification was performed using a BCA protein assay kit. The expression of apoptosis-related proteins and STING pathway-related proteins were detected according to the routine procedure of Western Blot. Antibodies used are shown in Table 3 of the Supplement; Important reagents are shown in Additional file 2: Tables S1 and S2.

### TdT-mediated dUTP nick end labeling (TUNEL) assays

Frozen sections, with a thickness of 5 μm, were fixed in 4% paraformaldehyde at a temperature of 4 ℃ for a duration of 24 h. Post-fixation, the sections were subjected to a blocking solution for 10 min at room temperature to subdue endogenous peroxidase activity. Subsequently, the sections were exposed to 50 μL of the TUNEL reaction solution for 1 h at 37 ℃, ensuring a light-free environment. Nuclei were stained with 4,6-diamidino-2-phenylindole (DAPI) for 15 min at room temperature, also in the absence of light. Finally, photomicrographs were captured using a fluorescence light scanning microscope.

### Bax fluorescent staining of myocardial tissue

Frozen sections of mouse heart were washed with PBS. The tissue samples were incubated with permeabilizate solution (0.1% Triton X-100) at room temperature for 30 min, then blocked at room temperature for 2 h, and further incubated with Bax antibody at 4 ℃ overnight, followed with another incubation with the secondary antibody at room temperature after washing, and DAPI nuclear staining. After washing, Tissue samples were scanned under fluorescence microscopy.

### Fluorescence assay of reactive oxygen species (ROS)

For the detection of ROS fluorescence, dihydroethidium (DHE) (Beyotime Biotech. Inc. Shanghai, China) was employed. myocardial tissue sections were thoroughly washed with PBS and subsequently incubated with a 5 μM concentration of DHE at 37 ℃ for 30 min. The specimens were then scanned with a fluorescence microscope and the intensity of fluorescence was quantitatively analyzed using ImageJ software.

### Determination of MDA contents, SOD and GSH activity

MDA contents and the activity of SOD and GSH in heart tissues and cultured cells were quantified using commercially available MDA, SOD, and GSH kits (Nanjing Jiancheng Bioengineering Institute. Nanjing, China). The obtained data was analyzed spectrophotometrically using an Agilent BioTek Gen5 spectrophotometer (Agilent Technologies, California, USA). MDA contents were measured at a wavelength of 532 nm, SOD at 450 nm, and GSH at 405 nm.

### Real-time quantitative PCR

Total RNA samples from mouse tissues and cultured cells were extracted using TRIzol reagent (ATG Biotechnology Co., Ltd., Nanjing, China). The RNA samples were used for reverse transcription and real-time quantitative PCR (qRT-PCR) following the protocol provided by TSINGKE (Beijing Qingdao Biotechnology Co., Ltd., China), SynScript®IIIRT SuperMix for qPCR (+ gDNA Remover) and 2 × TSINGKE®Master qPCR Mix (SYBR Green I). The Relative RNA levels were analyzed using the 2^-ΔΔct^ method, with GAPDH serving as an internal control. The primers sequences utilized for amplification can be found in Additional file 2: Table S4.

### Cell culture

The mouse cardiac muscle cell line (HL1) was acquired from Pricella (CL-0605, Procell Life Science &Technology Co., Ltd. China). HL1 cells were cultured in a high-glucose DMEM supplemented with 10% fetal bovine serum and penicillin (100 U/mL), and streptomycin (100 μg/mL) at a temperature of 37 ℃ with 5% CO_2_.

To establish the cellular model of hypoxia-reoxygenation, HL1 cells were cultivated in a hypoxic chamber comprising 1% O_2_, 5% CO_2_, and 94% N_2_ for a period of 12 h. Subsequently, the cells were reintroduced to normal oxygen conditions and reoxygenated in a complete medium for 6 h. This experimental procedure was utilized to simulate the physiological conditions of hypoxia and subsequent reoxygenation. The cells were divided into the following groups: an NC group, HR group, HR + As-IV group, HR + Ta-IIA group, and HR + Co group. Additionally, a solvent control group was established for both As-IV and Ta-IIA, namely the HR + DMSO group.

To generate an oxidative stress model, cells were stimulated with H_2_O_2_ (600 μM, a concentration determined from our previous experiments, Additional file 1: Fig. S3A) for a duration of 6 h. The cell samples were then divided into the following groups: NC group, H_2_O_2_ group, H_2_O_2_ + As-IV group, H_2_O_2_ + Ta-IIA group, and H_2_O_2_ + Co group.

### Cell viability analysis

The viability of the cells was determined by a CCK8 assay (Beyotime Biotech. Inc. Shanghai, China). HL1 cells were seeded in 96-well plates at a density of 10^4^ cells per well. Then, 10 μL of the CCK8 reagent was added to each 200 μL DMEM medium per well, and a blank control group was included. The cells were further incubated for an additional 40 min. The absorbance of each well was measured at a wavelength of 450 nm. The optimal concentrations of As-IV and Ta-IIA were optimized for subsequent experiments using cell viability assays. Co is a combination of As-IV and Ta-IIA with optimal concentrations.

### Flow cytometry analysis of annexin V-PI staining

Apoptosis was quantified using an Annexin V-APC/PI Apoptosis Kit (MULTISCIENCES, Hangzhou, China). Briefly, the cells were stained with Annexin V and propidium iodide (PI) after washed with PBS. Flow cytometry was performed using a CytoFLEX™ flow cytometer (Beckman Coulter, Inc., California, USA). Annexin V–PI − , Annexin V–PI + , Annexin V + PI − , and Annexin V + PI + staining represented viable cells, necrotic cells, early apoptotic cells, and late apoptotic cells, respectively.

### Analysis of ROS by flow cytometry

ROS production in cells was measured using an ROS assay kit (Beyotime Biotech. Inc. Shanghai, China). HL1 cells were washed with PBS for 3 times, and then incubated with the fluorescent probe 2,7-dichlorodihydrofluorescein diacetate (DCFH-DA) (10 μM) at 37 ℃ for 30 min. Stained cells were then acquired and analyzed with CytoFLEX™ flow cytometry to calculate mean fluorescence intensity and fluorescence curves.

### Binding affinity between Ta-IIA, As-IV, and STING by molecular docking

The canonical 2D structures of As-IV and Ta-IIA were retrieved from the PubChem database (https://pubchem.ncbi.nlm.nih.gov/) and converted into a 3D structures using ChemBio3D Ultra 14.0 (Cambridgesoft Inc. Cambridge, Massachusetts, United States). The 3D structures were then energetically minimized and saved in the MOL2 format. Additionally, the 3D structures of STING were generated using the PDB database (https://www.rcsb.org/). PyMOL 2.5.2 (http://autodock.scripps.edu/) was used to add hydrogen atoms and remove water molecules from the 3D structures, while the formats of As-IV, Ta-IIA, and STING were converted to a pdbqt file by AutoDock Tools 1.5.7 software (http://autodock.scripps.edu/). Finally, AutoDock Vina v.1.2.0 software (http://autodock.scripps.edu/) was used for molecular docking. The lower the binding energy, the more stable the ligand–protein binding conformation.

### Phosphorylated STING (p-STING) fluorescence staining of HL1 cells

The HL1 cells were seeded in 6-well plates with appropriate replicates and grouped accordingly. Following the assigned treatment, the cells were immobilized using 4% paraformaldehyde. Subsequently, they were incubated with a permeabilization solution (0.1% Triton X-100) for 30 min at room temperature. Afterwards, a blocking step was performed at room temperature for 2 h. The cells were then incubated overnight at 4 ℃ with the primary antibody against p-STING. After thorough washing, the cells were incubated at room temperature for 1 h with appropriate secondary antibodies. Meanwhile, DAPI was used to achieve nuclear staining. After blocking and washing, the cell samples were photographed and scanned with a fluorescence microscope.

### ***STING*** siRNA of HL1 cells

The HL1 cells were cultured at a density that ensured a confluence of 70%–80% at the time of transfection. The culture medium was replaced with serum-free medium. The siRNA and lipofectamine 3000 were diluted with Opti-MEM, respectively, incubated for 5 min, followed by thorough mixing in the same tube and incubation for 15 min. The prepared transfection solution was added to the cell culture plate drop by drop and gently shaken. After 6 h of siRNA transfection, the serum-free medium was replaced with complete medium. The transfection efficiency was confirmed after 24 h by performing Western blotting and qPCR to detect the levels of STING protein and gene expression respectively.

Following transfection, the experimental groups were set up as follows: negative control (NC) group, hypoxia-reoxygenation (HR) group, HR + *STING *siRNA group, HR + *STING *siRNA + As-IV group, HR + *STING *siRNA + Ta-IIA group, and HR + *STING *iRNA + Co group.

### Statistical analysis

GraphPad Prism v 8.2.1 was used for statistical analysis. The data were presented as mean (M) ± standard error of the mean (SEM). The comparison between the two groups was performed by unpaired t test. The significance level was set at p < 0.05.

## Results

### Ta-IIA and As-IV alleviates myocardium injury in MIRI

**Fig. 1:**
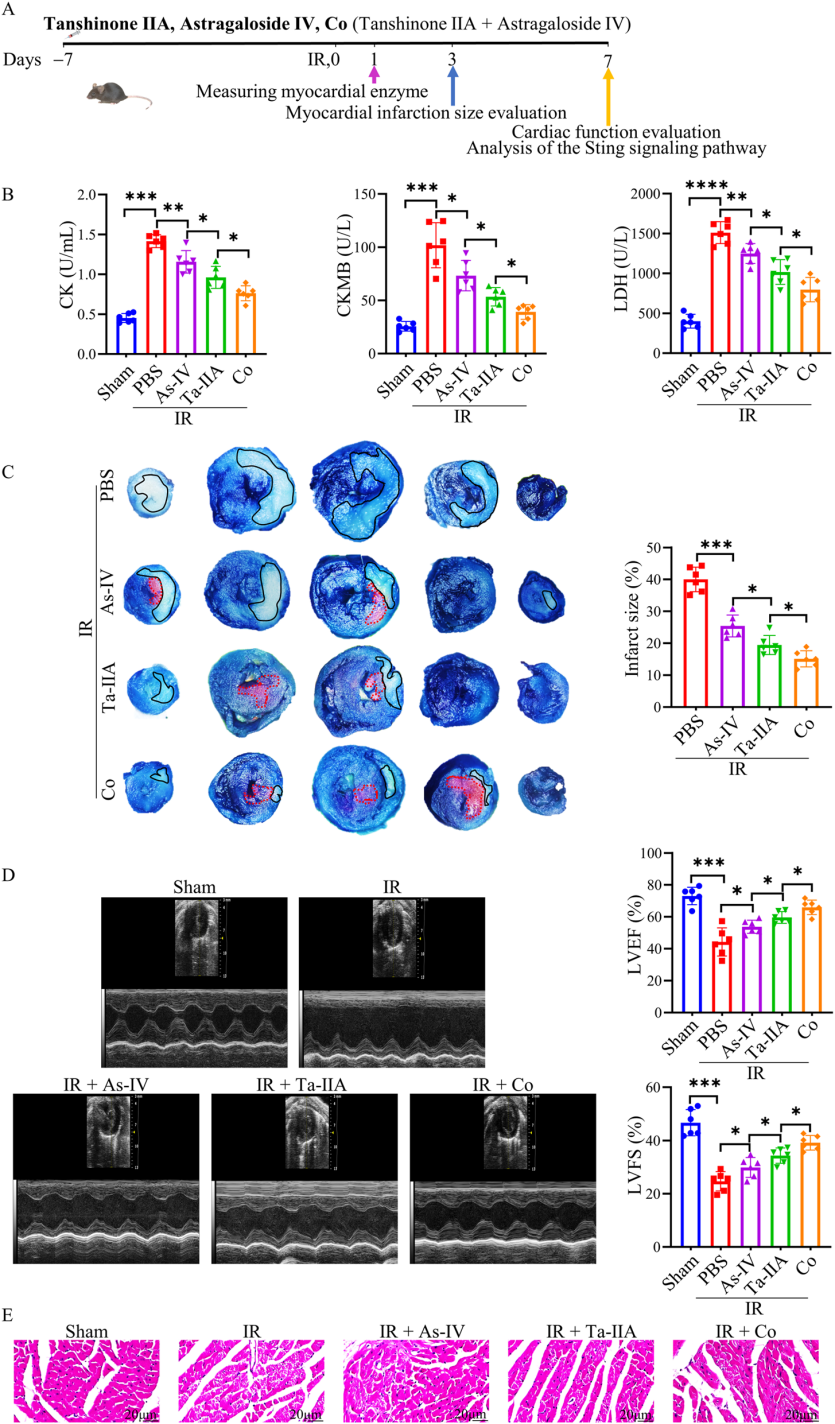
The protective effect of As-IV, Ta-IIA, and Co on the heart of MIRI mice. Ta-IIA treatment (10 mg/kg/day, i.p.), As-IV treatment (15 mg/kg/day, i.p.), Co (Ta-IIA 10 mg/kg/day + As-IV 15 mg/kg/day i.p.) treatment were given 7 days before reperfusion until tested after anesthesia. Myocardial enzymes and LDH were detected 1 day after surgery, EB/TTC double staining was detected 3 days after surgery, and cardiac function was detected by echocardiography and HE staining 7 days after surgery. **A** Experimental design of As-IV, Ta-IIA, and Co for in vivo cardioprotection in mice. **B** Serum CK, CKMB levels, and serum LDH levels. **C** Representative digital images of heart sections by Evans blue and TTC double staining, and the percentage of infarct area. The blue-stained portion indicates the normal region, the red-stained portion indicates the ischemic region, and the white portion indicates the infarcted region. The ratio of the infarct area of the largest heart section to the total area of the section was chosen to be the percentage of infarct area. **D** Representative M-mode echocardiographic images, and the quantitative analysis of the LVEF and LVFS. **E** Representative picture of HE staining of myocardial tissue. Data are presented as mean ± SEM. (n = 6 in each group). *p < 0.05, **p < 0.01, ***p < 0.001

To investigate the efficacy of the combined application (Co) of Ta-IIA and As-IV in treating MIRI, first, a MIRI mouse model was constructed, then the myocardial enzymes, infarct area, cardiac function and pathological changes were detected after the treatment of As-IV, Ta-IIA, and Co (Fig. 1A). Levels of CK, CKMB, and LDH were significantly higher in the IR group compared to the sham group (p < 0.001). However, As-IV, Ta-IIA, and Co were able to significantly reduce these parameters, with Co exhibiting the most substantial effect (p < 0.05, Fig. 1B). Additionally, As-IV, Ta-IIA, and Co were found to reduce the infarct area with white color in mouse hearts after MIRI (p < 0.001). This suggests that As-IV, Ta-IIA and Co could reduce myocardial infarction in mice, with Co showing the most significant effect in reducing the area (p < 0.05, Fig. 1C). To evaluate the heart phenotype, echocardiography was performed to determine myocardial contractility in mice. Mice treated with As-IV, Ta-IIA or Co showed markedly improved contractility compared with IR control, as evidenced by increased left ventricular ejection fraction (LVEF) and left ventricular fraction shortening (LVFS). Co treatment showed even better effect versus As-IV or Ta-IIA administration (p < 0.05, Fig. 1D). To further investigate the pathological changes after treatment, HE staining was applied on the tissue sections of heart. The data revealed that cardiomyocytes in the IR group were swollen, necrotic, and had irregular fascicular arrangements. However, after treatment with As-IV, Ta-IIA, and Co, there was a significant improvement in cell necrosis and myocardial bundle alignment. Notably, Co treatment resulted in the most significant improvement in myocardial muscle (Fig. 1E). Taken together, these data showed that As-IV and Ta-IIA effectively reduced myocardial enzymes, infarction area, pathological damage, and improved cardiac function. Furthermore, the combined application of As-IV and Ta-IIA showed the best results in treating MIRI.

In order to find more appropriate drug concentration for combination therapy, we applied different dosages of combined Ta-IIA and As-IV, and they are low dose (Ta-IIA 5 mg/kg/d + As-IV 10 mg/kg/d), medium dose (Ta-IIA 10 mg/kg/d + As-IV 15 mg/kg/d) and high dose (Ta-IIA 15 mg/kg/d + As-IV 20 mg/kg/d). The results showed that the medium-dose treatment was much better than the low-dose and high-dose treatments in improving both myocardial infarction size and myocardial enzymes without obvious side effects on mice (P < 0.05, Additional file 1: Fig. S1). Therefore, the medium dose (Ta-IIA 10 mg/kg/d + As-IV 15 mg/kg/d) was selected as an appropriate concentration in the following studies.

### Ta-IIA and As-IV reduced myocardial cell apoptosis, oxidative stress and inflammation caused by MIRI in vivo

**Fig. 2:**
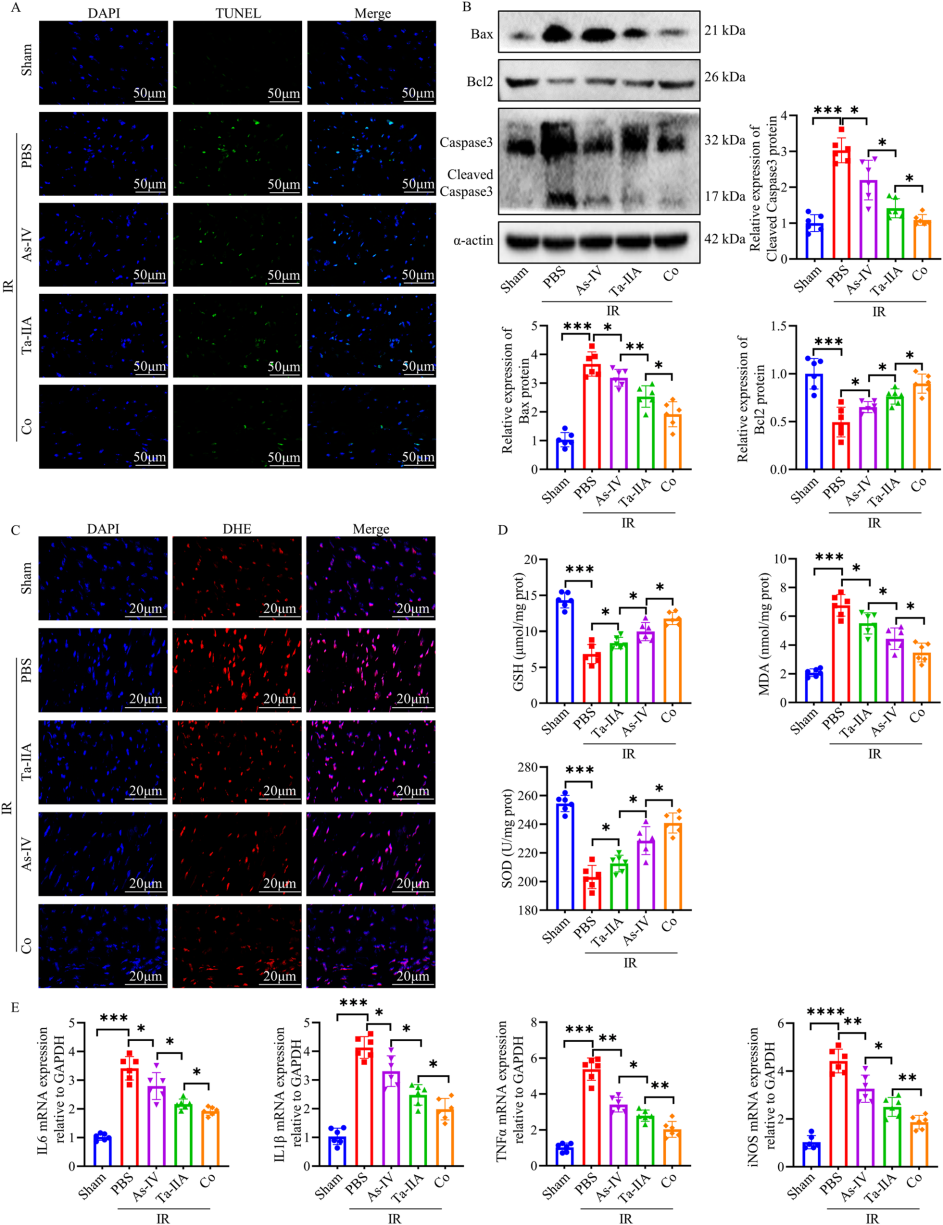
As-IV, Ta-IIA, and Co inhibited myocardial cell apoptosis, oxidative stress, and inflammatory response in MIRI. **A** Representative images of apoptotic cardiomyocytes. The apoptotic cells were detected by TUNEL (green), and the nuclei were detected by DAPI (blue). Scale bar = 50 μm. **B** Representative blots of the apoptosis-related proteins Bax, Bcl2, Caspase3, and Cleaved Caspase3 in myocardial tissue, as well as quantitative analysis of Bax, Bcl2, and Cleaved Caspase3. **C** Representative images of ROS content in cardiomyocytes. ROS was detected by DHE fluorescence staining (red), and nuclei were detected by DAPI (blue). scale bar = 20 μm. **D** Myocardial glutathione (GSH) activity, malondialdehyde(MDA) content, superoxide dismutase (SOD) activity. E. The mRNA quantification of cytokines IL-6, IL-1β, TNFα, iNOS in cardiomyocardial tissues. Data are presented as mean ± SEM. (n = 6 in each group). *p < 0.05, **p < 0.01, ***p < 0.001

To further investigate the mechanism how As-IV, Ta-IIA and Co display the protective effect on MIRI, we examined the apoptosis, oxidative stress, and inflammatory factors in different experimental groups. Firstly, As-IV and Ta-IIA and Co administration effectively decreased the amount of TUNEL^+^ cells compared with those in IR group, with Co exhibiting the most substantial reduction (P < 0.05, Fig. 2A**, **Additional file 1: Fig. S2A), which was further confirmed by reduced pro-apoptosis protein Bax and Cleaved Caspase3 and increased anti-apoptosis protein Bcl2 as showed by western blot or immunofluorescence staining, with Co exhibiting the most pronounced effect (P < 0.05, Fig. 2B, Additional file 1: Fig. S2B). Oxidative stress is one of the most important pathological mechanisms in inducing apoptosis during reperfusion injury. Therefore, we detected oxidative stress related indicators. The data showed that all three treatments reduced oxidative stress as evidenced by decreased DHE fluorescence intensity and MDA levels as well as enhanced GSH and SOD activities (P < 0.05, Fig. 2C, D). What’s more, Ta-IIA was more effective than As-IV, while Co was more effective than Ta-IIA in reducing oxidative stress (P < 0.05, Fig. 2C, D). Furthermore, given that interleukin-6 (IL-6), interleukin-1β (IL-1β), tumor necrosis factor α (TNFα) are commonly pro-inflammatory cytokines, and iNOS serves as a prevalent inflammation-inducing factor[29], the assessment of mRNA levels of these cytokines can reflect changes in the inflammatory response. Notably, As-IV exhibited the ability to reduce the mRNA expression of IL-6, IL-1β, TNFα, and iNOS in myocardial tissue induced by IR (P = 0.035, 0.012, 0.0012, 0,0038, respectively), while Ta-IIA demonstrated a better ability to reduce above inflammatory factors compared to As-IV, and Co showed an even stronger capability compared to Ta-IIA (P < 0.05, Fig. 2E). The above results suggested that As-IV and Ta-IIA can significantly reduce apoptosis, oxidative stress, and inflammation in MIRI compared to IR group, and Co treatment showed more potent effect than As-IV and Ta-IIA alone.

### Ta-IIA and As-IV protected HL1 cells against apoptosis, oxidative stress, and inflammation in vitro

**Fig. 3:**
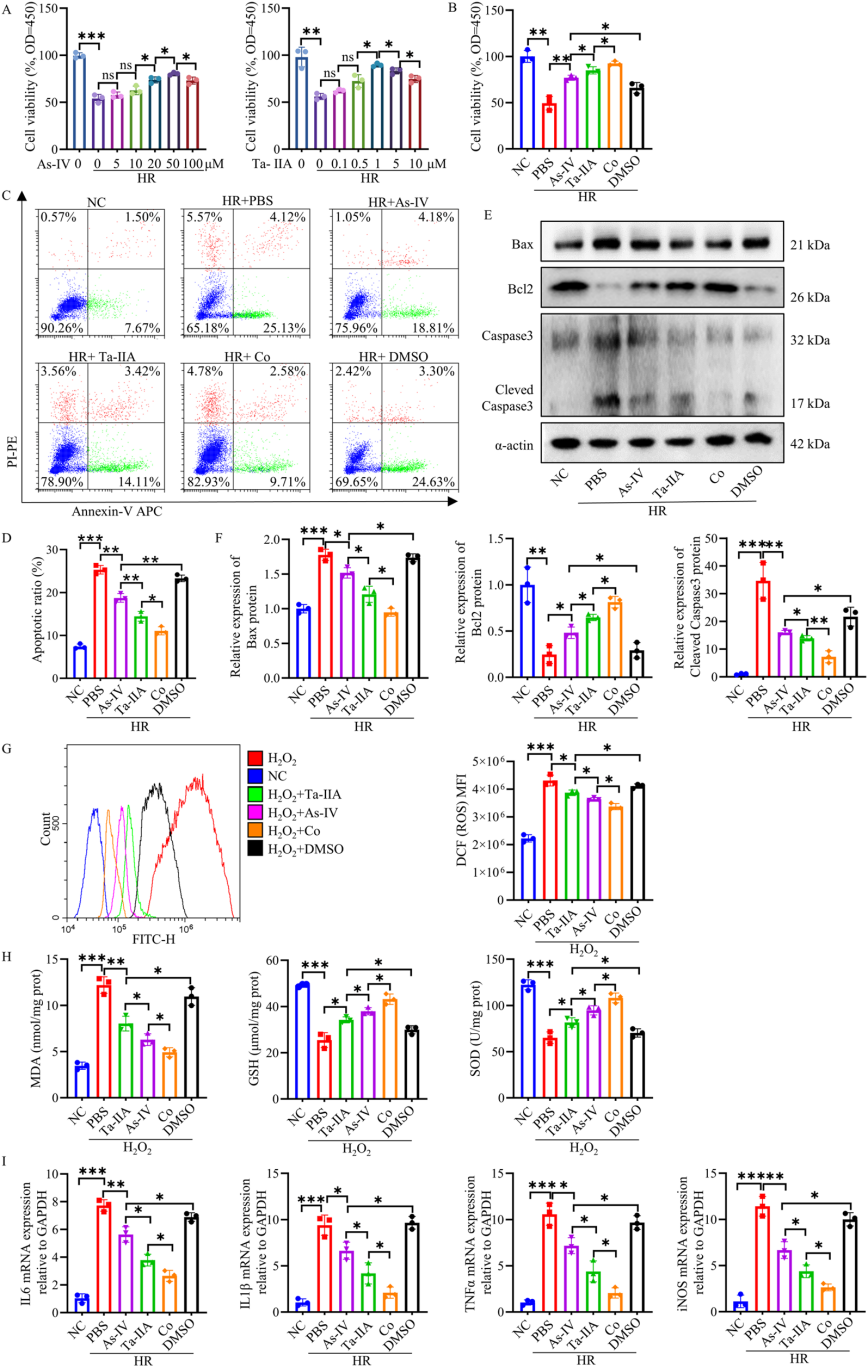
Effects of As-IV and Ta-IIA on HR-induced apoptosis, oxidative stress, and inflammation in HL1 cells. **A** As-IV and Ta-IIA treatment dose- dependently increased the viability of HR- injured HL1 cells. **B** The increase in cell activity with Co treatment was greater in HR-injured HL1 cells than in those treated with Ta-IIA and As-IV. Ta-IIA was 1 μm, and As-IV was 50 μm. Co was a combination of Ta-IIA1 μm and As-IV 50 μm. **C**. Representative results of apoptosis rates measured in HL1 cells using flow cytometry. **D** Quantitative analysis of the apoptosis rate in (**C**). **E** Representative western blots of Bax, Bcl2, Cleaved Caspase3. F. The quantitative analysis of protein expression in (**E**). **G** Representative picture of the fluorescence curve of DCF in HL1 cells and the quantitative analysis of DCF mean fluorescence intensity. This was detected by flow cytometry. H. Cellular MDA content, GSH activity, SOD activity. I. Quantitative analysis of cytokines IL-6, IL-1β, TNFα and iNOS mRNA in HL1 cells. Data are presented as mean ± SEM. (n = 3 in each group). *p < 0.05, **p < 0.01, ***p < 0.001, ns = not statistically significant

​To further validate our observed results in vivo, we constructed an in vitro model using HL1 cells to detect the apoptosis rates, oxidative stress response, and expression of inflammatory factors in a Hypoxia/Reoxygenation** (**HR) model. We first treated HR-induced HL1 cells with different concentrations of As-IV and Ta-IIA, and the best cell viability was found under the conditions of 50 μM for As-IV and 1 μM for Ta-IIA (Fig. 3A). Therefore, subsequent experiments were carried out at these concentrations. It demonstrated that Co treatment showed even greater enhancement in cell viability compared to treatment with either As-IV or Ta-IIA alone (P < 0.05, Fig. 3B). We then detected the effect of As-IV, Ta-IIA and Co on cell apoptosis by Annexin V-APC/PI apoptosis detecting kit. HL1 cells treated with Co, As-IV and Ta-IIA showed a significant reduction in early apoptosis compared to HR-induced cells (P < 0.01, Fig. 3C, D). Subsequently, the expression of apoptosis-related proteins in HL1 cells were also investigated, and the levels of pro-apoptosis proteins Bax and Cleaved Caspase3 were higher and anti-apoptosis protein Bcl2 was lower in the HR group compared to the NC group. However, after treatment with As-IV, Ta-IIA and Co, the expression of these proteins was reversed (P < 0.05, Fig. 3E, F), suggesting that As-IV, Ta-IIA and Co have the strong ability to inhibit the apoptosis of HR-induced HL1 cells, and Co exhibited a superior anti-apoptosis effect compared to As-IV and Ta-IIA. Furthermore, we applied H_2_O_2_- induced cell injury model. Compared to H_2_O_2_ treatment, additional adding Ta-IIA, As-IV or Co shifted the DCF fluorescence curve to the left and decreased the mean fluorescence intensity (P < 0.05, Fig. 3G), suggesting the decrease of oxidative stress in the cells. Consistent with in vivo data, Ta-IIA, As-IV and Co significantly decreased MDA, and enhanced GSH and SOD with Co having the most significant restorative effect (P < 0.05, Fig. 3H). In addition, Co was more effective than Ta-IIA and As-IV in reducing inflammatory factors including IL-6, IL-1β, TNFα and iNOS induced by HR, which is consistent with in vivo results (P < 0.05, Fig. 3I). In summary, the combined application of As-IV and Ta-IIA improved HR- and H_2_O_2_-induced HL1 apoptosis, inflammation, and oxidative stress more than either As-IV or Ta-IIA alone.

### Ta-IIA and As-IV perform the cardioprotective effect via targeting STING in MIRI

**Fig. 4:**
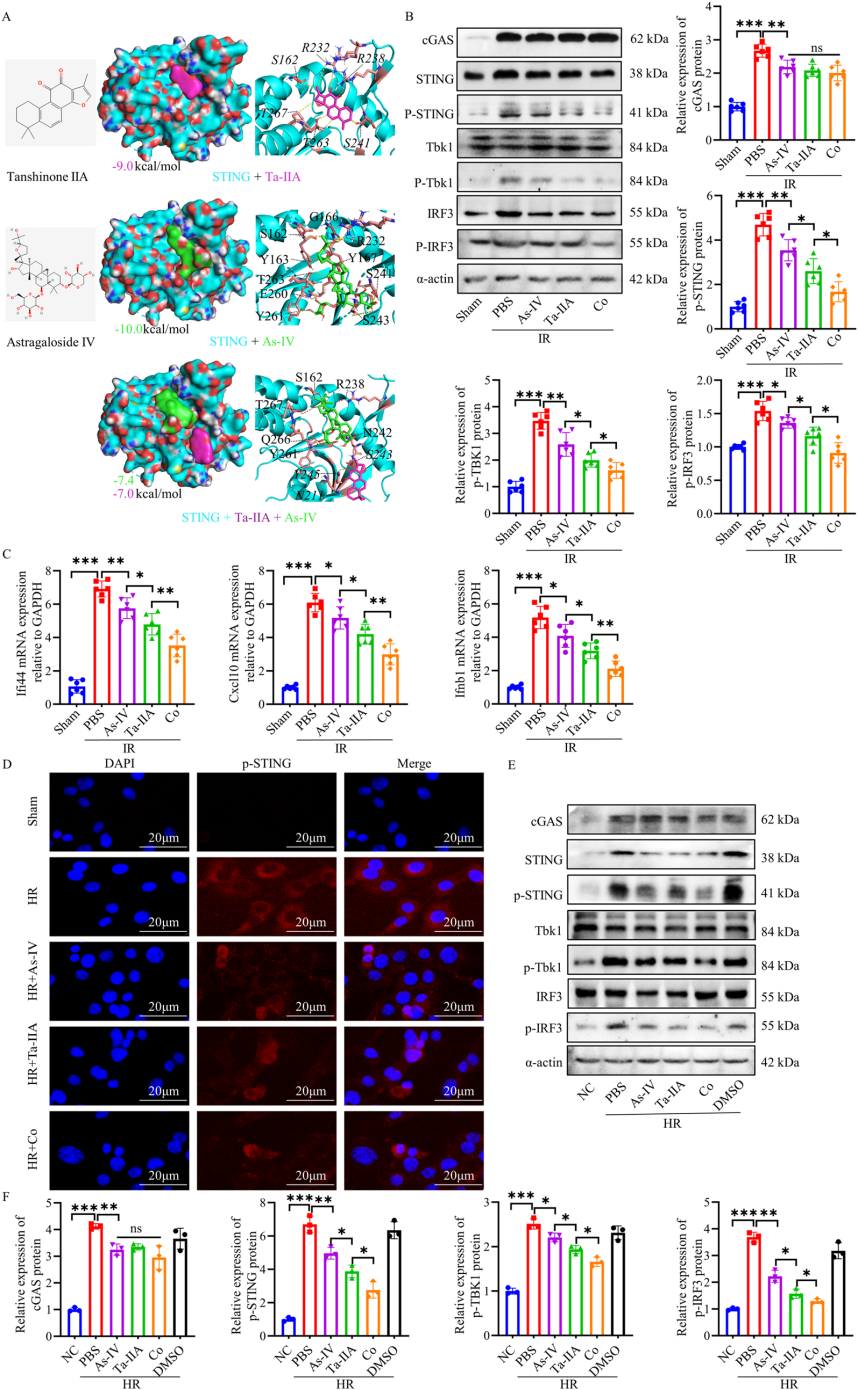
As-IV and Ta-IIA inhibited the STING pathway. **A** Chemical structure and representative simulations of the binding to the STING protein for As-IV and Ta-IIA. **B** Representative western blots and quantification of STING pathway-associated proteins in myocardial tissue. **C** Quantification of mRNA expression of the cytokines Ifi44, Cxcl10 and Ifnb1 downstream of the STING pathway caused by MIRI. **D** Representative immunofluorescence staining of p-STING in HL1 cells. The p-STING proteins were stained red, and the nuclei were detected by DAPI (blue). Scale bar = 20 μm. **E** Representative western blots of STING pathway-associated proteins in HR-induced HL1 cells. **F** The quantitative analysis of protein expression in (**E**). Data are presented as mean ± SEM. (n = 6 in each group in vivo; n = 3 in each group in vitro). *p < 0.05, **p < 0.01, ***p < 0.001, ns = not statistically significant

To further investigate the targets and pathways regulated by Ta-IIA, As-IV and Co in mitigating MIRI, we conducted a screening of proteins that can potentially interact with Ta-IIA and As-IV. Molecular docking experiments indicated that both Ta-IIA and As-IV exhibit a favorable affinity towards STING. Figure 4A illustrated the optimal interaction sites for Ta-IIA and As-IV binding to STING, respectively. The binding energy ranged from − 7.3 kcal/mol to the optimal value of -10 kcal/mol, and ranged from − 6.6 kcal/mol to the best value of -9.0 kcal/mol for the binding of STING with As-IV and Ta-IIA, respectively (Fig. 4A**, **Tables 1 and 2). These findings highlighted the robust binding affinity of As-IV and Ta-IIA towards STING. Subsequently, we attempted to dock As-IV and Ta-IIA together with the STING protein and compared them with individual docking. It was found that both As-IV and Ta-IIA can bind to the STING protein simultaneously with high binding affinities to STING (− 7.4 kcal/mol and − 7.0 kcal/mol). On the other hand, compared to individual molecule docking, the binding position and interaction site of two drugs with STING protein have also changed during simultaneous molecule docking (Fig. 4A). When As-IV was individually docked to the STING protein, it interacted with the binding sites G166, R232, Y167, S241, S243, Y261, E260, T263, Y163 and S162, while Ta-IIA interacted with R232, R238, S241, T263, T267 and S162. When co-docked with STING, As-IV interacted with the sites S162, R238, N242, Y261, Q266 and T267, and Ta-IIA interacted with sites S243, N211 and Y245.

**Table 1:** Different binding sites and binding energies of Astragaloside IV and STING

Mode	Affinity	Dist from best mode	
	(kcal/mol)	rmsd l.b	rmsd u.b
1	− 10.0	0.000	0.000
2	− 9.9	1.194	2.148
3	− 9.9	1.304	1.923
4	− 8.9	4.134	9.861
5	− 8.6	3.820	9.884
6	− 8.5	2.939	9.123
7	− 8.4	4.750	9.046
8	− 8.3	3.681	10.427
9	− 8.2	4.910	10.033
10	− 8.1	3.604	5.107
11	− 8.1	4.517	10.169
12	− 7.8	4.418	8.188
13	− 7.8	3.470	5.885
14	− 7.7	5.392	10.279
15	− 7.7	1.972	2.949
16	− 7.6	21.821	25.631
17	− 7.4	4.389	6.973
18	− 7.3	21.619	24.475
19	− 7.3	21.078	24.510
20	− 7.3	5.554	13.448

**Table 2:** Different binding sites and binding energies of tanshinone II a and STING

Mode	Affinity	Dist from best mode	
	(kcal/mol)	rmsd l.b	rmsd u.b
1	− 9.0	0.000	0.000
2	− 8.8	1.972	4.368
3	− 8.3	1.553	2.569
4	− 8.3	1.908	5.931
5	− 8.1	3.170	4.493
6	− 8.0	4.296	6.797
7	− 8.0	2.712	3.767
8	− 7.7	2.073	6.425
9	− 7.6	4.535	8.420
10	− 7.5	5.270	8.775
11	− 7.5	3.157	7.190
12	− 7.4	7.282	10.264
13	− 7.3	2.183	6.459
14	− 7.2	5.073	7.048
15	− 7.1	3.332	6.890
16	− 7.0	4.069	6.896
17	− 7.0	12.439	16.072
18	− 6.8	5.149	7.430
19	− 6.7	3.848	6.219
20	− 6.6	15.234	17.677

Next, we analyzed the expression levels of pertinent proteins within the STING pathway in myocardial tissue. The results revealed an upregulation of cGAS, p-STING, p-TBK1, and p-IRF3 in the IR group, indicating the activation of the STING pathway. However, treatments with As-IV, Ta-IIA, and Co resulted in a reduction in the expression of p-STING, p-TBK1 and p-IRF3 induced by IR (P < 0.05, Fig. 4B). Additionally, these treatments also downregulated the mRNA expression of the downstream cytokines Ifi44, Cxcl10, and Ifnb1 [12, 30] (P < 0.05, Fig. 4C), highlighting the inhibitory effects of As-IV, Ta-IIA, and Co on the STING signaling pathway. Intriguingly, the expression of IR-induced cGAS, an upstream protein of the STING signaling pathway, was not attenuated by treatment with As-IV, Ta-IIA, and Co. This indicates that the targets of As-IV, Ta-IIA, and Co may not be resided upstream of STING, but rather the STING protein itself. Notably, Co exhibited more strong inhibition on the phosphorylation of STING and its downstream proteins, surpassing the effects of As-IV and Ta-IIA (P < 0.05, Fig. 4B; P < 0.01, Fig. 4C).

Additional experiments were also conducted in vitro to investigate the alterations of STING signaling following HR induction in HL1 cells. The results demonstrated that HR induction leads to an increase in the fluorescence intensity of p-STING (Fig. 4D**)**, as well as an increase of the expression of proteins related to the STING pathway including p-STING, p-TBK1 and p-IRF3 (P < 0.001, Fig. 4E, F). Furthermore, the downstream cytokines Ifi44, Cxcl10, and Ifnb1 were also exhibited an upward trend (P < 0.01, Additional file 1: Fig. S3B). However, As-IV, Ta-IIA, and Co were observed to reduce these HR-induced alterations, with Co displaying the most significant reduction (P < 0.05, Fig. 4D–F**, **Additional file 1: Fig. S3B). These findings were consistent with the results obtained from in vivo experiments, highlighting the inhibitory effect of As-IV, Ta-IIA, and Co on the STING pathway. Remarkably, the combination of two ingredients showed a more pronounced inhibitory effect.

### ​The STING signaling pathway is involved in the cardioprotective effects of Ta-IIA, As-IV and Co on MIRI

**Fig. 5:**
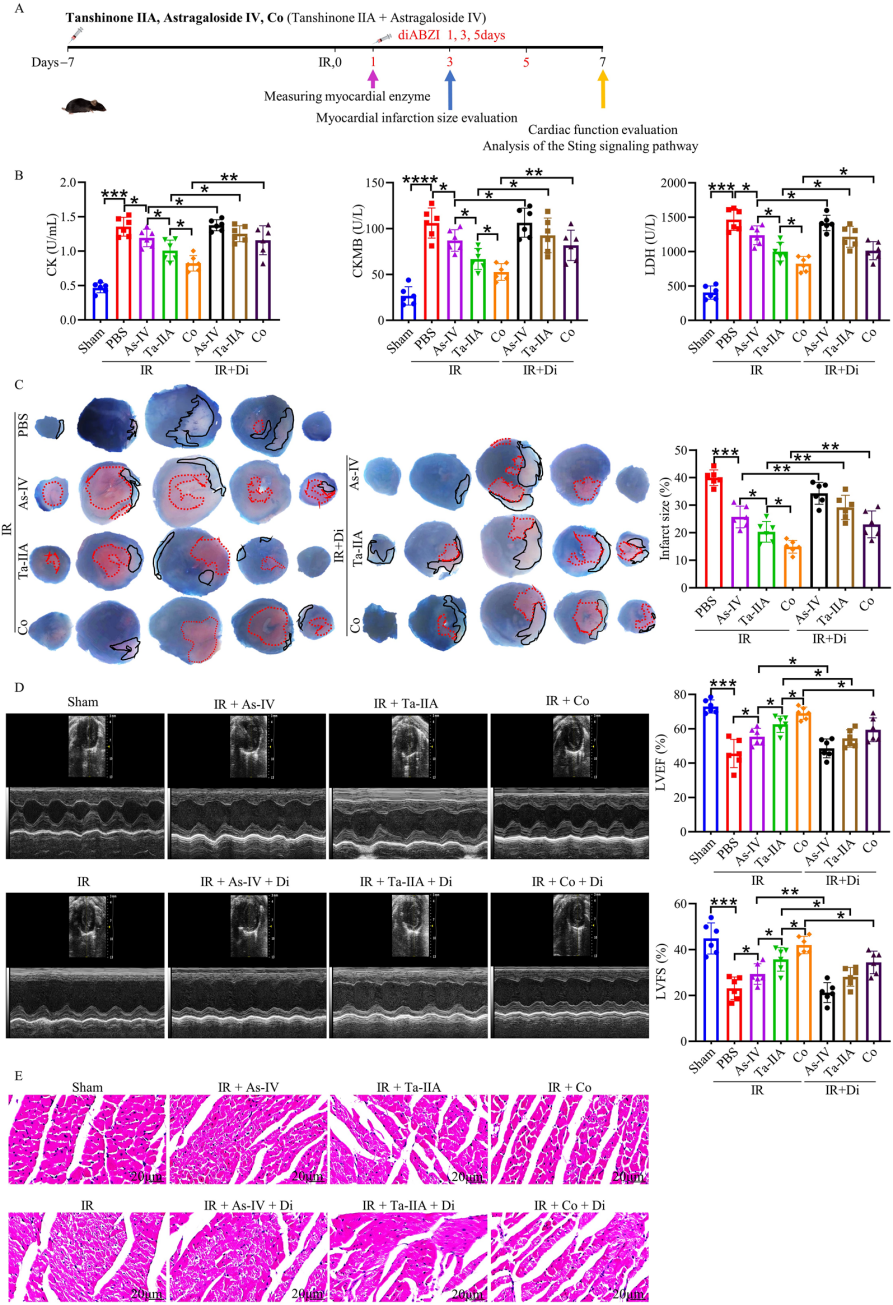
STING agonist treatment attenuated the myocardial protection of Ta-IIA, As-IV, and Co on MIRI mice. Ta-IIA treatment (10 mg/kg/d, i.p.), As-IV treatment (15 mg/kg/day, i.p.), Co (Ta-IIA 10 mg/kg/day + As-IV 15 mg/kg/day i.p.) treatment were given 7 days before reperfusion until tested after anesthesia. DiabZI (3 mg/kg, i.v.) was administered once every other day for a total of three times following reperfusion surgery. **A** Experimental procedures for As-IV, Ta-IIA and Co in mice after the addition of agonists. **B** Serum CK, CKMB levels, and serum LDH levels. **C** Representative digital images of heart sections by Evans blue and TTC double staining, and the percentage of infarct area. The blue-stained portion indicates the normal region, the red-stained portion indicates the ischemic region, and the white portion indicates the infarcted region. The ratio of the infarct area of the largest heart section to the total area of the section was chosen to be the percentage of infarct area. **D** Representative M-mode echocardiographic images, and the quantitative analysis of the LVEF and LVFS. **E** Representative picture of HE staining of myocardial tissue. Data are presented as mean ± SEM. (n = 6 in each group). *p < 0.05, **p < 0.01, ***p < 0.001

To further validate the cardioprotective effects of As-IV, Ta-IIA, and Co in MIRI mice by inhibiting STING pathway, IR mice were administered with diABZI, a STING agonist, intravenously after treatment with As-IV, Ta-IIA, and Co, followed by subsequent measurements of myocardial enzymes, infarct size, and cardiac function (Fig. 5A). The analysis of myocardial enzymes showed that As-IV, Ta-IIA, and Co could not further reduce blood CK, CKMB, and LDH after intravenous injection of the STING agonist (P < 0.05, Fig. 5B). Similar observations were obtained in terms of the infarct area (P < 0.01), left ventricular function (LVEF, LVFS, P < 0.05), and cardiac pathological damage (Fig. 5C–E). These findings suggested that the cardioprotective effects of As-IV, Ta-IIA, and Co are mediated through the inhibition of STING.

### Ta-IIA, As-IV mediated anti-apoptotic, antioxidant and anti-inflammatory effects via suppressing STING signaling pathway on MIRI

**Fig. 6:**
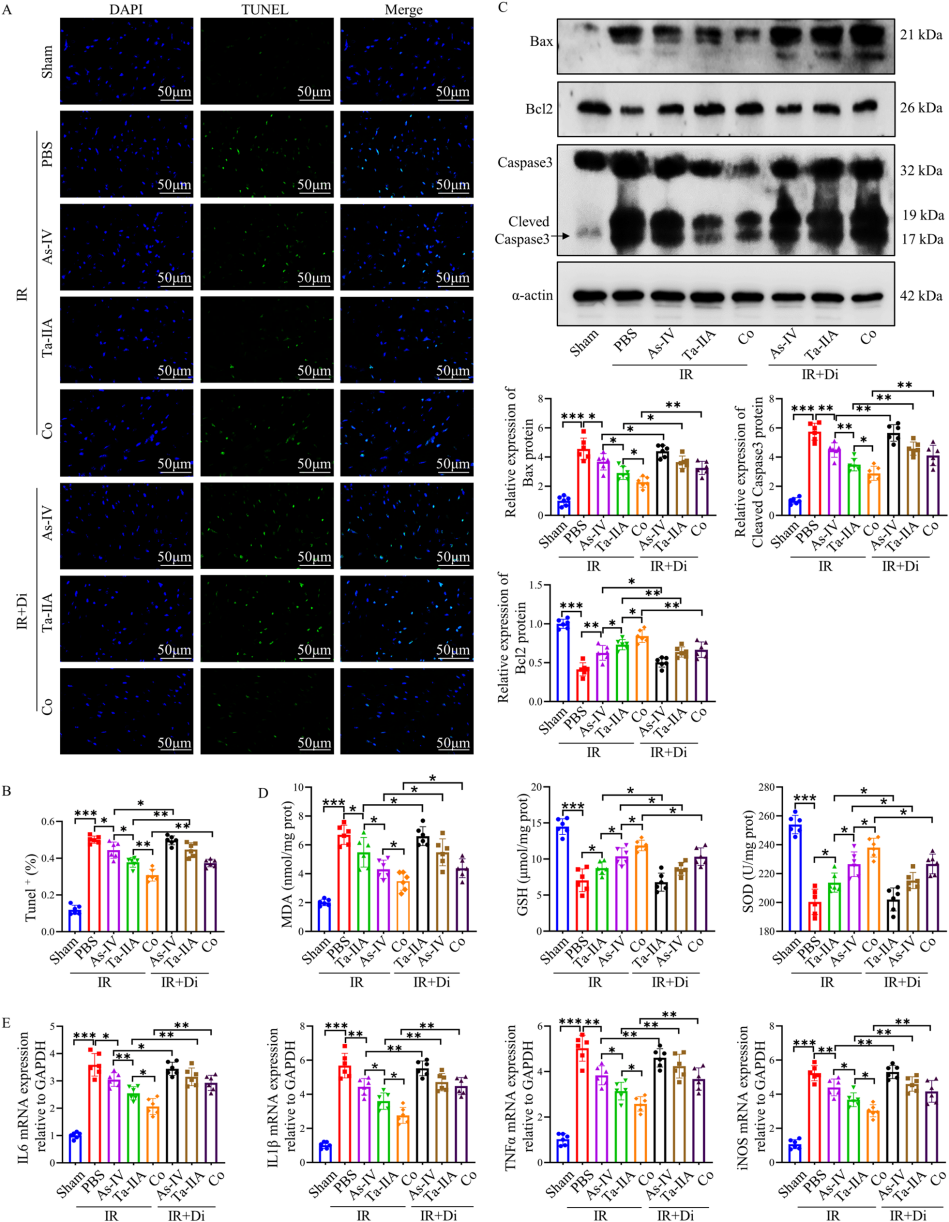
The anti-apoptotic, antioxidant and anti-inflammatory effects of Ta-IIA, As-IV and Co were weakened by diabZI. **A** Representative images of apoptotic cardiomyocytes. The apoptotic cells were detected by TUNEL (green), and the nuclei were detected by DAPI (blue). Scale bar = 50 μm. **B** The quantitative analysis of Tunel^+^ cells of (**A**). **C** Representative blots of the apoptosis-related proteins in myocardial tissue, as well as quantitative analysis of Bax, Bcl2, and Cleaved Caspase3. **D** Myocardial MDA content, GSH activity, SOD activity. E. The mRNA quantification of cytokines IL-6, IL-1β, TNFα, iNOS in cardiomyocardial tissues. Data are presented as mean ± SEM. (n = 6 in each group). *p < 0.05, **p < 0.01, ***p < 0.001

In order to explore the potential anti-apoptotic, antioxidant, and anti-inflammatory effects mediated by As-IV, Ta-IIA, and Co, primarily by inhibiting STING, we evaluated the levels of apoptosis, oxidative stress markers, and inflammatory cytokine in myocardial tissue. However, upon intravenous administration of the STING agonist, treatment with As-IV, Ta-IIA, and Co did not exhibit significant reductions in the number of TUNEL^+^ cells (Fig. 6A , B, P< 0.05**)** or the expression of pro-apoptotic proteins Bax and Cleaved Caspase3 induced by IR (P < 0.05, Fig. 6C and Additional file 1: Fig. S4A). These data suggested that the anti-apoptotic effects mediated by As-IV, Ta-IIA, and Co are impeded following STING activation. Furthermore, diABZI administration offset the anti-oxidative stress effect at certain extent as evidenced by increased DHE fluorescence and MDA, and decreased GSH and SOD compared with As-IV, Ta-IIA, or Co treatment alone (Additional file 1: Fig. S4B, and Fig. 6D,  P< 0.05). Additionally, the anti-inflammatory effects of As-IV, Ta-IIA, and Co were also attenuated as showed by enhanced mRNA levels of IL-6, IL-1β, TNFα, iNOS following the administration of the STING agonist (P < 0.05, Fig. 6E). Summing up, these findings suggest that As-IV, Ta-IIA, and Co primarily exert their anti-apoptotic, antioxidant, and anti-inflammatory effects mainly through STING inhibition.

### The STING signaling pathway is crucial for mediating the anti-apoptotic, anti-oxidant, and anti-inflammatory effects of Ta-IIA, As-IV, and Co on HL1 cells after HR

**Fig. 7:**
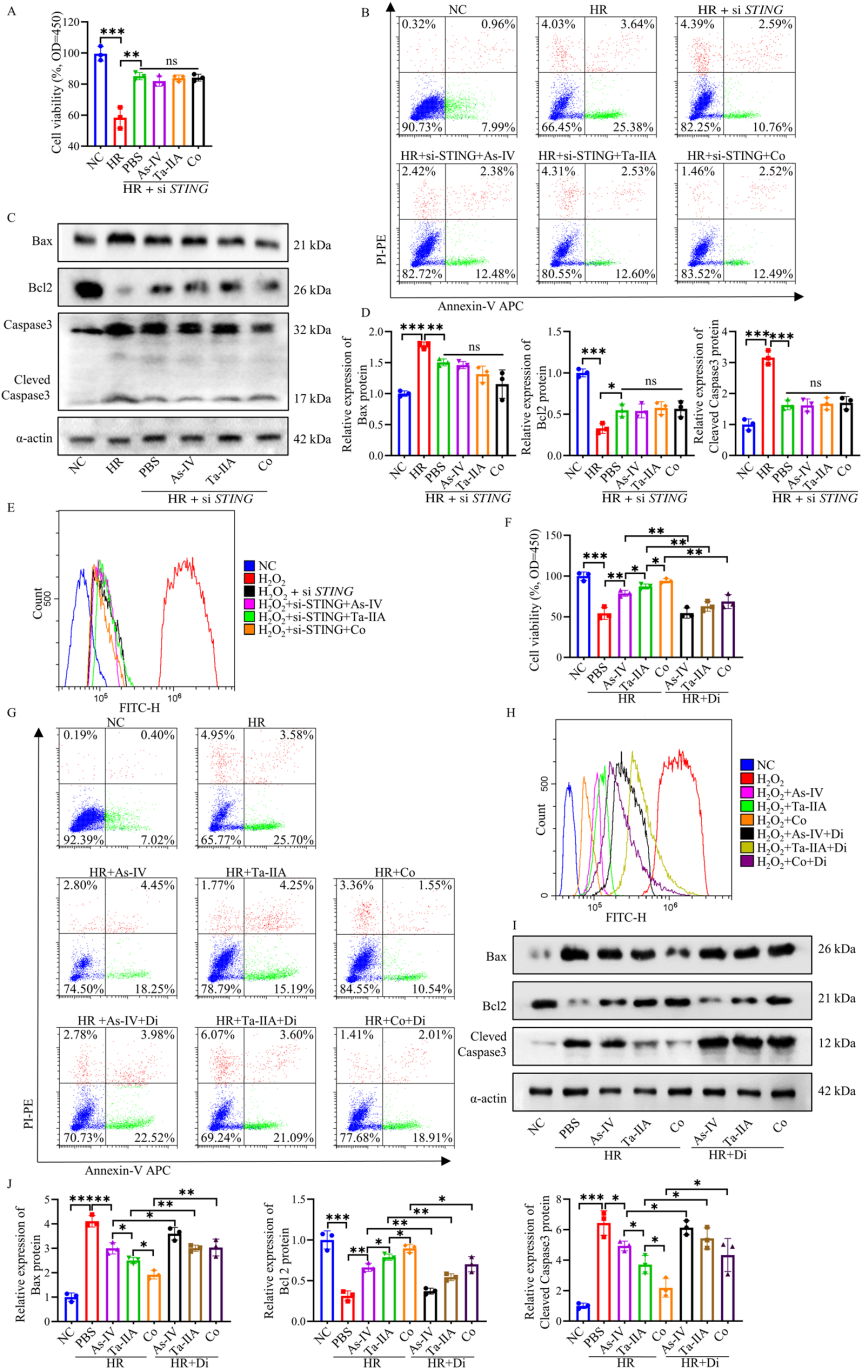
The STING pathway is critical for Ta-IIA, As-IV, and Co effect on HR-induced HL1 cells. The effects of Ta-IIA, As-IV and Co on cell activity, apoptosis, oxidative stress and inflammation were detected in HL1 cells after *STING* siRNA and diabZI administration, respectively. **A** HL1 cells *STING* siRNA cell activity after Ta-IIA, As-IV and Co treatment. **B** Representative results of apoptosis rates measured in HL1 cells using flow cytometry. C. Representative western blots of Bax, Bcl2, Cleaved Caspase3. **D** The quantitative analysis of protein expression in (**C**). **E** Representative picture of the fluorescence curve of DCF in HL1 cells. **F** Cell activity of Ta-IIA, As-IV and Co treatment after diabZI administration. **G** Representative pictures of apoptosis rates of HL1 cells measured by flow cytometry. **H** Representative diagram of the DCF fluorescence curve in HL1 cells. **I** Representative western blotting of apoptosis-associated proteins. **J** Quantitative analysis of protein expression in (**I**). Data are presented as mean ± SEM. (n = 3 in each group). *p < 0.05, **p < 0.01, ***p < 0.001, ns = not statistically significant

The protective effects of As-IV, Ta-IIA and Co via inhibiting STING signaling pathway were further validated in vitro HL1 cell models. To this end, HL1 cells were transfected with *STING* siRNA (HL1^△*STING*^), resulting in a significant decrease of STING expression in protein and mRNA levels (P < 0.05, Additional file 1: Fig. S5A, B). We first identified the effect of *STING* knock-down on cell viability. It showed that the treatment of As-IV, Ta-IIA or Co did not alter the viability of HL1^△*STING*^ cells in a HR experimental model (Fig. 7A**)**. Then the apoptosis status was evaluated after treatment by PI/Annexin-V staining. HL1^△*STING*^ cells with As-IV, Ta-IIA or Co administration demonstrated similar frequencies of early- and late-apoptosis cells, and similar levels of Bax, Bcl2, and Caspase3 proteins compared with HL1^*wt*^ cells, further confirming that As-IV, Ta-IIA or Co perform their cardioprotective effect by targeting STING signaling pathway (ns, Fig. 7B**–**D, Additional file 1: Fig. S5C). Similarly, following *STING* knocking down, As-IV, Ta-IIA, and Co failed to display the effect of anti-oxidative stress and inhibiting the production of inflammatory factors (ns, Fig. 7E and Additional file 1: Fig. S5D–F). Furthermore, when HR-induced HL1 cells were treated with diABZI, the protective effects mediated by As-IV, Ta-IIA, and Co including increased cell viability, low apoptosis rate, low oxidative stress and inflammatory factor content were significantly attenuated (P < 0.05, Fig. 7F–J, Additional file 1: Fig. S6A–D). These results suggest that As-IV, Ta-IIA, and Co can regulate apoptosis, oxidation, and inflammation by inhibiting the STING signaling pathway, thereby protecting cardiomyocytes.

## Discussion

MIRI exerts a significant impact on the development and prognosis of cardiovascular disease. Implementing protective measures targeted towards MIRI can effectively mitigate cardiomyocyte mortality, restore cardiac function, and ultimately enhance the therapeutic outcomes and prognosis of cardiovascular disease [31]. The utilization of Ta-IIA and As-IV has demonstrated notable improvements in cardiovascular disease, including MIRI treatment [21, 32]. However, the efficacy and underlying mechanism of the combined therapy involving As-IV and Ta-IIA for the treatment of MIRI have yet to be investigated. Within our study, we sought to examine the therapeutic effectiveness of co-application of these two drugs and found that this approach yielded greater efficacy in mitigating MIRI compared to treatment with either As-IV or Ta-IIA alone. Additionally, this combination exhibited enhanced potency in suppressing STING phosphorylation and its downstream STING signaling pathway, consequently yielding stronger anti-apoptotic, antioxidant and anti-inflammatory effects.

In the realm of traditional Chinese medicine, the principle of "Replenishing Qi and activating blood" is one of the important treatment principles for the treatment of cardiovascular diseases [33]. The utilization of therapeutic medications embodying the "Replenishing Qi and activating blood" effect ameliorates cardiovascular symptoms [34]. Numerous studies have embarked on exploring the mechanism of "Replenishing Qi and activating blood" in treating cardiovascular and cerebrovascular diseases [35–37]. However, these studies mainly focus on the overall study of prescriptions, and neither investigate specific small molecule drugs, nor the effect differences between small molecule drugs [38]. In order to further study the specific mechanism of "Replenishing Qi and activating blood" in the treatment of MIRI, the As-IV (the main pharmaceutical ingredient in the representative drug *Astragalus membranaceus* (Fisch.) Bunge of "Replenishing Qi" category [24]) and Ta-IIA (the main pharmaceutical ingredient in the representative drug *Salvia miltiorrhiza* Bunge of the "Activating blood" category [22]) were selected in our study.

Previous studies have reported that the combined application of Ta-IIA and As-IV can promote the angiogenesis of endothelial cell-like cells [39], attenuate atherosclerotic plaque vulnerability [40], and reduce hypoxia-induced cardiomyocytes injury [32]. Our study was primarily focused on the effect of Co on MIRI and found that compared to As-IV or Ta-IIA alone, Co exhibited superior ability in reducing the area of myocardial infarction, reducing myocardial enzymes levels, and restoring myocardial contractility.

During the process of reperfusion, the abrupt reintroduction of oxygen to the mitochondria generates ROS, which exacerbates mitochondrial damage and triggers an inflammatory response, ultimately resulting in cell death [4, 41]. Mitochondrial dysfunction plays a crucial role in the development of MIRI [42]. Numerous cardioprotective measures focus on mitochondria to reduce oxidative stress, inflammatory responses, and alleviate apoptosis [43–45]. Both Ta-IIA and As-IV have demonstrated protective effects in MIRI by reducing cardiomyocyte apoptosis [25, 46], oxidative stress [25, 47], and inflammation [27, 48]. Our experiments demonstrated that As-IV and Ta-IIA effectively decrease apoptosis, oxidative stress, and inflammation in MIRI cardiomyocytes. What's more, we have discovered that Co possesses a more potent anti-apoptotic, antioxidant, and anti-inflammatory effect compared to using As-IV or Ta-IIA individually.

Molecular docking analysis of As-IV, Ta-IIA, and STING were performed to forecast the interaction patterns and binding affinities. The results revealed that both As-IV and Ta-IIA exhibit strong binding affinity to the STING protein, whether docked individually or simultaneously. In the context of simultaneous molecule docking, the binding location and interaction site of the two drugs with the STING protein have undergone alterations when juxtaposed with individual molecule docking. During simultaneous docking, the binding sites of As-IV to STING have changed from G166, R232, Y167, S241, S243, Y261, E260, T263, Y163, and S162 to S162, R238, N242, Y261, Q266, and T267. Meanwhile, Ta-IIA has transformed from R232, R238, S241, T263, T267, and S162 to S243, N211, and Y245. Therefore, it appears to remain within a substantial structural domain, albeit with alterations to the loci of interaction. Among these residues, R232, R238, S162, E260, and T263 are common sites for STING activation [9]. Additionally, STING can be phosphorylated starting from residue S243 [49].

Moreover, it has been demonstrated that As-IV alleviates immunosuppression by modulating the STING signaling pathway in disease infected by virus [50]. In cardiovascular disease, mitochondrial dysfunction leads to the release of mtDNA into the cytoplasm, triggering cGAS, subsequently activating the STING signaling pathway and eliciting the release of inflammatory mediators that exacerbate cellular damage and inflammatory response [11, 13].

In our experiments, the expression of cGAS, p-STING, and downstream p-TBK1 and p-IRF3 proteins exhibited an increase in both IR mice and HR-induced HL1 cells. This demonstrated that the activation of STING signaling was also initiated in MIRI. We observed that As-IV, Ta-IIA, and Co significantly reduced the expression of phosphorylated STING, TBK1, and IRF3, but not the expression of cGAS. This indicated that these drugs effectively inhibit the phosphorylation of STING protein and, in turn, STING signaling pathway activation. Additionally, we also found that Co exhibits a stronger inhibition on p-STING and STING signaling pathways compared to As-IV and Ta-IIA. This suggested that Co possesses superior therapeutic efficacy, enhancing the suppressive effect of As-IV and Ta-IIA on STING, thereby augmenting the protection against MIRI.

On the other hand, the efficacy of As-IV, Ta-IIA, and Co in improving myocardial infarction, myocardial enzymes, and restoring cardiac contractile function was diminished upon the addition of STING agonist (diABZI) in in vivo experiments. The data provided further evidence that As-IV, Ta-IIA and Co exert their cardioprotective effects against MIRI by suppressing the STING pathway.

The activation of the STING pathway also induces a plethora of ROS, which can damage the DNA, proteins and lipids of cells, leading to oxidative stress [51]. In our study, we found a tight link between increased oxidative stress and activation of the STING pathway, which is important in the development and progression of MIRI. However, the application of As-IV, Ta-IIA and Co efficiently inhibited oxidative stress in myocardial caused by STING phosphorylation. Furthermore, the STING agonist (diABZI) can counteract the anti-oxidative stress effects of As-IV, Ta-IIA and Co.

The STING pathway induces the release of interferon, which promotes apoptosis by inhibiting the cell cycle, increasing the permeability of the mitochondrial membrane, activating key proteins in apoptosis pathway, and activating the DNA damage response [52]. In our study, we have observed a significant increase in STING pathway-associated proteins and cell apoptosis in MIRI-induced myocardium and HR-induced HL1 cells. However, the effect was reduced with As-IV, Ta-IIA and Co treatment, whereas it was reverted with diABZI.

The activated STING pathway produces signaling molecules such as interferon and NF-κB, which in turn activates immune cells to participate in the inflammatory reactions, promotes the release of inflammatory factors, and triggers the inflammatory response [9]. In our investigation, the inflammatory cytokines in MIRI or HR-induced HL1 cells were increased along with the activation of the STING pathway, but were decreased upon As-IV, Ta-IIA and Co treatment. Nevertheless, diABZI reversed the anti-inflammatory effect of As-IV, Ta-IIA and Co.

Taken together, our results demonstrated that As-IV, Ta-IIA, and Co exerts anti-apoptotic, antioxidant, and anti-inflammatory effect, and inhibit the STING signaling pathway, ultimately suppressed MIRI. However, there exist certain limitations in our study. First, we did not elucidate how the combination of As-IV and Ta-IIA enhances the inhibitory effect on STING phosphorylation. Second, the combination of As-IV and Ta-IIA may potentially affect myocardial cells through other type of cells present in the myocardial tissue, which is a possible aspect that needs to be further investigated.

## Conclusion

Our study found that the combination of As-IV and Ta-IIA was more effective than As-IV or Ta-IIA alone, as it contributes to the improvement of the area of myocardial infarction, reducing myocardial enzyme levels, and promoting myocardial contractility recovery. Moreover, Co has enhanced anti-apoptotic, antioxidant and anti-inflammatory effects through intensified suppression of STING signaling both in vivo and in vitro. This study provides evidence for the co-application of As-IV and Ta-IIA in the treatment of MIRI, provides research strategies and theoretical support for the treatment of MIRI by traditional Chinese medicines such as "Replenishing Qi" and "Activating blood", and provides an important foundation and inspiration for further investigation of the MIRI mechanism.

### Supplementary Information

**Additional file 1: Figure S1.** Screening of drug concentrations for combined therapy. Low dose (Ta-IIA 5 mg/kg/d + As-IV 10 mg/kg/d), medium dose (Ta-IIA 10 mg/kg/d + As-IV15 mg/kg/d), high dose (Ta-IIA 15 mg/kg/d + As-IV 20 mg/kg/d) were used. The three concentrations were given 7 days before reperfusion until postoperative detection. Myocardial enzymes and LDH were detected at 1 day after operation, and EB/TTC double staining was detected at 3 days after operation. **A** Representative digital images of heart sections by Evans blue and TTC double staining, and the percentage of infarct area. The blue-stained portion indicates the normal region, the red-stained portion indicates the ischemic region, and the white portion indicates the infarcted region. The ratio of the infarct area of the largest heart section to the total area of the section was chosen to be the percentage of infarct area. **B** Serum CK, CKMB levels, and serum LDH levels. Data are presented as mean ± SEM. (n = 6 in each group). *p < 0.05, **p < 0.01, ***p < 0.001. **Figure S2**. **A** The quantitative analysis of Tunel^+^ cells of myocardial tissue in Fig. 2A. Data are presented as mean ± SEM. (n = 6 in each group). *p < 0.05, **p < 0.01, ***p < 0.001. **B** Representative immunofluorescence staining of Bax protein expression in cardiomyocytes of MIRI treated by Ta-IIA, As-IV, and Co. The Bax proteins were stained red, and the nuclei were detected by DAPI (blue). **Figure S3**. **A** Cell activity of HL1 cells incubated with different concentrations of H_2_O_2_ for 6 h. **B** Quantification of mRNA expression of the cytokines Ifi44, Cxcl10 and Ifnb1 downstream of the STING pathway in HL1 cells induced by HR. Data are presented as mean ± SEM. (n = 3 in each group). *p < 0.05, **p < 0.01, ***p < 0.001, ns = not statistically significant. **Figure S4**. **A** Representative immunofluorescence staining of Bax protein expression in MIRI cardiomyocytes treated with Ta-IIA, As-IV and Co after agonist addition. The Bax proteins were stained red, and the nuclei were detected by DAPI (blue). **B** Representative images of ROS content in cardiomyocytes. ROS was detected by DHE fluorescence staining (red), and nuclei were detected by DAPI (blue). scale bar = 20 μm. **Figure S5**. The STING pathway crucial for Ta-IIA, As-IV and Co effects on HR-induced HL1 cells. **A** The efficiency of *STING* siRNA was confirmed by western blot. **B** The efficiency of *STING* siRNA was confirmed by qPCR. C. Quantitative analysis of the apoptosis rate of HL1 cells after *STING* siRNA. **D** Quantitative analysis of the DCF mean fluorescence intensity of HL1 cells after *STING* siRNA. **E** Cellular MDA content, GSH activity, SOD activity of HL1 cells after *STING* siRNA. **F** Quantitative analysis of cytokines IL-6, IL-1β, TNFα and iNOS mRNA in *STING* siRNA HL1 cells. Data are presented as mean ± SEM. (n = 3 in each group). *p < 0.05, **p < 0.01, ***p < 0.001, ns = not statistically significant. **Figure S6**. **A** Quantitative analysis of the apoptosis rate of HL1 cells after diabZI administration. **B** Quantitative analysis of the DCF mean fluorescence intensity of HL1 cells after diabZI administration. **C** Cellular malondialdehyde(MDA) content, glutathione (GSH) activity, superoxide dismutase (SOD) activity of HL1 cells after diabZI administration. **D** Quantitative analysis of cytokines IL-6, IL-1β, TNFα and iNOS mRNA in HL1 cells diabZI administration. Data are presented as mean ± SEM. (n = 3 in each group). *p < 0.05, **p < 0.01, ***p < 0.001.

**Additional file 2: Table S1**. List of key chemicals. **Table S2**. List of commercial assays. **Table S3**. List of antibodies. **Table S4**. Primers used in qPCR assay.

## Data Availability

The original data presented in the study have been presented in the article and supplementary material, and further data can be requested by directly contacting the corresponding authors.

## Abbreviations

Ta-IIA: Tanshinone IIA
As-IV: Astragaloside IV
Co: Combination
TCM: Traditional Chinese medicine
MI: Myocardial infarction
MIRI: Myocardial ischemia and reperfusion injury
cGAS: Cyclic GMP-AMP synthase
cGAMP: 2′3’-Cyclic GMP-AMP
STING: Stimulator of interferon genes
TBK 1: TANK-binding kinase 1
IRF 3: Interferon regulatory factor 3
mtDNA: Mitochondrial DNA
IFNβ: Interferon-β
ifi 44: Interferon induced protein 44
ifnb 1: Interferon beta 1
CXCL 10: Chemokine (C-X-C motif) ligand 10
IL-6: Interleukin-6
IL-1β: Interleukin-1β
TNF α: Tumor necrosis factor α
iNOS: Inducible nitric oxidesynthase
ROS: Reactive oxygen species
NF-κB: Nuclear factor kappa-light-chain-enhancer of activated B cells
CK: Creatine kinase
CKMB: Creatine kinase muscle/brain subtype
LDH: Lactate dehydrogenase
EB: Evans blue
TTC: 2,3,5-Triphenyl tetrazolium chloride
LVEF: Left ventricular ejection fraction
LVFS: Left ventricular fractional shortening
HE: Hematoxylin eosin
TUNEL: TdT-mediated dUTP nick end labeling
DAPI: 40,6-Diamidino-2-phenylindole
Bcl 2: B-cell lymphoma-2
Bax: BCL 2 associated X
DHE: Dihydroethidium
DCFH-DA: 2,7-Dichlorodihydrofluorescein diacetate
DCF: 2',7'-Dichlorofluorescein
MDA: Malondialdehyde
SOD: Superoxide dismutase
GSH: Glutathione

## References

[1] Oseran AS, Yeh RW (2022). Time to treatment in ST-segment elevation myocardial infarction: identifying dangerous delays or diminishing returns?. JAMA.

[2] Chen J, Huang Q, Li J (2023). Panax ginseng against myocardial ischemia/reperfusion injury: a review of preclinical evidence and potential mechanisms. J Ethnopharmacol.

[3] Schafer A, Konig T, Bauersachs J (2022). Novel therapeutic strategies to reduce reperfusion injury after acute myocardial infarction. Curr Probl Cardiol.

[4] Ibanez B, Heusch G, Ovize M (2015). Evolving therapies for myocardial ischemia/reperfusion injury. J Am Coll Cardiol.

[5] Sun L, Wu J, Du F (2013). Cyclic GMP-AMP synthase is a cytosolic DNA sensor that activates the type I interferon pathway. Science.

[6] Wu J, Sun L, Chen X (2013). Cyclic GMP-AMP is an endogenous second messenger in innate immune signaling by cytosolic DNA. Science.

[7] Zhang C, Shang G, Gui X (2019). Structural basis of STING binding with and phosphorylation by TBK1. Nature.

[8] Liu S, Cai X, Wu J (2015). Phosphorylation of innate immune adaptor proteins MAVS, STING, and TRIF induces IRF3 activation. Science.

[9] Zhang Z, Zhou H, Ouyang X (2022). Multifaceted functions of STING in human health and disease: from molecular mechanism to targeted strategy. Signal Transduct Target Ther.

[10] Hopfner KP, Hornung V (2020). Molecular mechanisms and cellular functions of cGAS-STING signalling. Nat Rev Mol Cell Biol.

[11] Oduro PK, Zheng X, Wei J (2022). The cGAS-STING signaling in cardiovascular and metabolic diseases: Future novel target option for pharmacotherapy. Acta Pharm Sin B.

[12] King KR, Aguirre AD, Ye Y-X (2017). IRF3 and type I interferons fuel a fatal response to myocardial infarction. Nat Med.

[13] Xiong Y, Leng Y, Tian H (2023). Decreased MFN2 activates the cGAS-STING pathway in diabetic myocardial ischaemia-reperfusion by triggering the release of mitochondrial DNA. Cell Commun Signal.

[14] Yan M, Li Y, Luo Q (2022). Mitochondrial damage and activation of the cytosolic DNA sensor cGAS-STING pathway lead to cardiac pyroptosis and hypertrophy in diabetic cardiomyopathy mice. Cell Death Discov.

[15] Li JK, Song ZP, Hou XZ (2023). Scutellarin ameliorates ischemia/reperfusion injury-induced cardiomyocyte apoptosis and cardiac dysfunction via inhibition of the cGAS-STING pathway. Exp Ther Med.

[16] Liao Y, Cheng J, Kong X (2020). HDAC3 inhibition ameliorates ischemia/reperfusion-induced brain injury by regulating the microglial cGAS-STING pathway. Theranostics.

[17] Li FH, Huang XL, Wang H (2020). Protective effect of Yi-Qi-Huo-Xue decoction against ischemic heart disease by regulating cardiac lipid metabolism. Chin J Nat Med.

[18] Liang P, Mao L, Ma Y (2021). A systematic review on Zhilong Huoxue Tongyu capsule in treating cardiovascular and cerebrovascular diseases: pharmacological actions, molecular mechanisms and clinical outcomes. J Ethnopharmacol.

[19] Maione F, De Feo V, Caiazzo E (2014). Tanshinone IIA, a major component of Salvia milthorriza Bunge, inhibits platelet activation via Erk-2 signaling pathway. J Ethnopharmacol.

[20] Wu L, Fan Z, Gu L (2023). QiShenYiQi dripping pill alleviates myocardial ischemia-induced ferroptosis via improving mitochondrial dynamical homeostasis and biogenesis. J Ethnopharmacol.

[21] Luo Z, Liu Y, Zhao Z (2020). Effects of Astragalus injection and Salvia Miltiorrhiza injection on serum inflammatory markers in patients with stable coronary heart disease: a randomized controlled trial protocol. Trials.

[22] Rao S, Lin Y, Lin R (2022). Traditional Chinese medicine active ingredients-based selenium nanoparticles regulate antioxidant selenoproteins for spinal cord injury treatment. J Nanobiotechnology.

[23] Jin HJ, Xie XL, Ye JM (2013). TanshinoneIIA and cryptotanshinone protect against hypoxia-induced mitochondrial apoptosis in H9c2 cells. PLoS ONE.

[24] Zhang C, Li L, Hou S (2021). Astragaloside IV inhibits hepatocellular carcinoma by continually suppressing the development of fibrosis and regulating pSmad3C/3L and Nrf2/HO-1 pathways. J Ethnopharmacol.

[25] Mei M, Tang F, Lu M (2015). Astragaloside IV attenuates apoptosis of hypertrophic cardiomyocyte through inhibiting oxidative stress and calpain-1 activation. Environ Toxicol Pharmacol.

[26] Peng Q, Wang J, Han M (2023). Tanshinone IIA inhibits osteoclastogenesis in rheumatoid arthritis via LDHC-regulated ROS generation. Chin Med.

[27] Su Y, Yin X, Huang X (2022). Astragaloside IV ameliorates sepsis-induced myocardial dysfunction by regulating NOX4/JNK/BAX pathway. Life Sci.

[28] Li Y, Yu P, Fu W (2021). Ginseng-Astragalus-oxymatrine injection ameliorates cyclophosphamide-induced immunosuppression in mice and enhances the immune activity of RAW264.7 cells. J Ethnopharmacol.

[29] Paramita Pal P, Sajeli Begum A, Ameer Basha S (2023). New natural pro-inflammatory cytokines (TNF-alpha, IL-6 and IL-1beta) and iNOS inhibitors identified from *Penicillium polonicum* through in vitro and in vivo studies. Int Immunopharmacol.

[30] Huijser E, Bodewes ILA, Lourens MS (2022). Hyperresponsive cytosolic DNA-sensing pathway in monocytes from primary Sjogren's syndrome. Rheumatology (Oxford).

[31] Yang Y, Shao M, Yao J (2023). Neocryptotanshinone protects against myocardial ischemia-reperfusion injury by promoting autolysosome degradation of protein aggregates via the ERK1/2-Nrf2-LAMP2 pathway. Phytomedicine.

[32] Wang D, Liu Y, Zhong G (2017). Compatibility of Tanshinone IIA and Astragaloside IV in attenuating hypoxia-induced cardiomyocytes injury. J Ethnopharmacol.

[33] Zhang M, Sun MY, Chen QT (2022). The efficacy of Yiqi Huoxue method in treating coronary artery disease after percutaneous coronary intervention: a meta-analysis in accordance with PRISMA guideline. Medicine (Baltimore).

[34] Wang Y, Yang JH, Wan HT (2021). Efficacy of Yangyin Yiqi Huoxue Granule () in treatment of ischemic stroke patients with qi-yin deficiency and blood stasis syndrome: a randomized, double-blind, multicenter, phase-2 clinical trial. Chin J Integr Med.

[35] Chen M, Zhong G, Liu M (2023). Integrating network analysis and experimental validation to reveal the mitophagy-associated mechanism of Yiqi Huoxue (YQHX) prescription in the treatment of myocardial ischemia/reperfusion injury. Pharmacol Res.

[36] Gao H, Peng C, Wu L (2021). Yiqi-Huoxue granule promotes angiogenesis of ischemic myocardium through miR-126/PI3K/Akt axis in endothelial cells. Phytomedicine.

[37] Wu H, Gao H, Gao S (2019). A Chinese 4-herb formula, Yiqi-Huoxue granule, alleviates H(2)O(2)-induced apoptosis by upregulating uncoupling protein 2 in H9c2 cells. Phytomedicine.

[38] Wang Y, Liu X, Zhang W (2022). Synergy of "Yiqi" and "Huoxue" components of QishenYiqi formula in ischemic stroke protection via lysosomal/inflammatory mechanisms. J Ethnopharmacol.

[39] Li Z, Zhang S, Cao L (2018). Erratum: Tanshinone IIA and Astragaloside IV promote the angiogenesis of mesenchymal stem cell-derived endothelial cell-like cells via upregulation of Cx37, Cx40 and Cx43. Exp Ther Med.

[40] Wang N, Zhang X, Ma Z (2020). Combination of Tanshinone IIA and Astragaloside IV attenuate atherosclerotic plaque vulnerability in ApoE(-/-) mice by activating PI3K/AKT signaling and suppressing TRL4/NF-kappaB signaling. Biomed Pharmacother.

[41] Salameh A, Dhein S, Mewes M (2020). Anti-oxidative or anti-inflammatory additives reduce ischemia/reperfusions injury in an animal model of cardiopulmonary bypass. Saudi J Biol Sci.

[42] Tian J, Zheng Y, Mou T (2023). Metformin confers longitudinal cardiac protection by preserving mitochondrial homeostasis following myocardial ischemia/reperfusion injury. Eur J Nucl Med Mol Imaging.

[43] Xiang M, Lu Y, Xin L (2021). Role of oxidative stress in reperfusion following myocardial ischemia and its treatments. Oxid Med Cell Longev.

[44] Peter S (2021). Mitochondrial dysfunction as part of an inflammatory intermediate phenotype that drives premature ageing. J Intern Med.

[45] Maneechote C, Kerdphoo S, Jaiwongkam T (2023). Chronic pharmacological modulation of mitochondrial dynamics alleviates prediabetes-induced myocardial ischemia-reperfusion injury by preventing mitochondrial dysfunction and programmed apoptosis. Cardiovasc Drugs Ther.

[46] Xu L, He D, Wu Y (2022). Tanshinone IIA inhibits cardiomyocyte apoptosis and rescues cardiac function during doxorubicin-induced cardiotoxicity by activating the DAXX/MEK/ERK1/2 pathway. Phytomedicine.

[47] Song T, Yao Y, Wang T (2017). Tanshinone IIA ameliorates apoptosis of myocardiocytes by up-regulation of miR-133 and suppression of Caspase-9. Eur J Pharmacol.

[48] Xu J, Tian Z, Li Z (2023). Puerarin-Tanshinone IIA Suppresses atherosclerosis inflammatory plaque via targeting succinate/HIF-1alpha/IL-1beta axis. J Ethnopharmacol.

[49] Guo Y, Zhang XN, Su S (2023). beta-adrenoreceptor-triggered PKA activation negatively regulates the innate antiviral response. Cell Mol Immunol.

[50] Song K, Yu JY, Li J (2023). Astragaloside IV regulates cGAS-STING signaling pathway to alleviate immunosuppression caused by PRRSV infection. Viruses.

[51] Zou M, Ke Q, Nie Q (2022). Inhibition of cGAS-STING by JQ1 alleviates oxidative stress-induced retina inflammation and degeneration. Cell Death Differ.

[52] Cho SJ, Pyo S (2010). Interferon-gamma enhances the apoptosis of macrophages under trophic stress through activation of p53 and the JAK1 pathway. Arch Pharm Res.

