# Supplementary material for: The combination of Tanshinone IIA and Astragaloside IV attenuates myocardial ischemia–reperfusion injury by inhibiting the STING pathway
Source: Chin Med. 2024 Feb 28;19:34. doi: 10.1186/s13020-024-00908-y (PMC10900662; doi:10.1186/s13020-024-00908-y)
Supplement: Supplementary file 1 — Additional file 1: Figure S1. Screening of drug concentrations for combined therapy. Low dose (Ta-IIA 5 mg/kg/d + As-IV 10 mg/kg/d), medium dose (Ta-IIA 10 mg/kg/d + As-IV15 mg/kg/d), high dose (Ta-IIA 15 mg/kg/d + As-IV 20 mg/kg/d) were used. The three concentrations were given 7 days before reperfusion until postoperative detection. Myocardial enzymes and LDH were detected at 1 day after operation, and EB/TTC double staining was detected at 3 days after operation. A Representative digital images of heart sections by Evans blue and TTC double staining, and the percentage of infarct area. The blue-stained portion indicates the normal region, the red-stained portion indicates the ischemic region, and the white portion indicates the infarcted region. The ratio of the infarct area of the largest heart section to the total area of the section was chosen to be the percentage of infarct area. B Serum CK, CKMB levels, and serum LDH levels. Data are presented as mean ± SEM. (n = 6 in each group). *p < 0.05, **p < 0.01, ***p < 0.001. Figure S2. A The quantitative analysis of Tunel+ cells of myocardial tissue in Fig. 2A. Data are presented as mean ± SEM. (n = 6 in each group). *p < 0.05, **p < 0.01, ***p < 0.001. B Representative immunofluorescence staining of Bax protein expression in cardiomyocytes of MIRI treated by Ta-IIA, As-IV, and Co. The Bax proteins were stained red, and the nuclei were detected by DAPI (blue). Figure S3. A Cell activity of HL1 cells incubated with different concentrations of H2O2 for 6 h. B Quantification of mRNA expression of the cytokines Ifi44, Cxcl10 and Ifnb1 downstream of the STING pathway in HL1 cells induced by HR. Data are presented as mean ± SEM. (n = 3 in each group). *p < 0.05, **p < 0.01, ***p < 0.001, ns = not statistically significant. Figure S4. A Representative immunofluorescence staining of Bax protein expression in MIRI cardiomyocytes treated with Ta-IIA, As-IV and Co after agonist addition. The Bax proteins were stained red, and [file 13020_2024_908_MOESM1_ESM.pdf]

Supplementary Figure S1

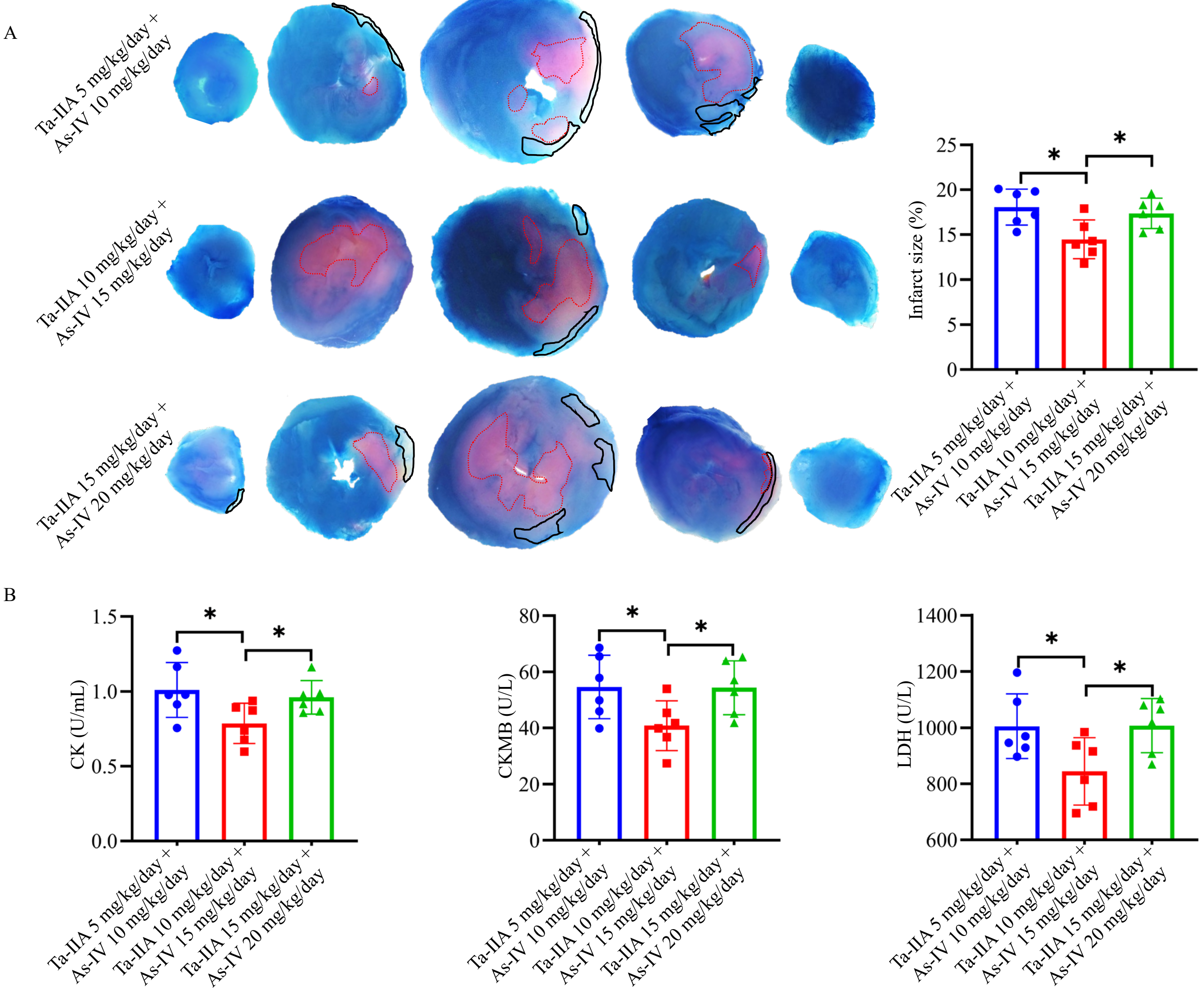

Supplementary Figure S1. Screening of drug concentrations for combined therapy. Low dose (Ta-IIA 5 mg/kg/d + As-IV10 mg/kg/d), medium dose (Ta-IIA 10 mg/kg/d + As-IV15 mg/kg/d), high dose (Ta-IIA 15 mg/kg/d + As-IV20 mg/kg/d) were used. The three concentrations were given 7 days before reperfusion until postoperative detection. Myocardial enzymes and LDH were detected at 1 day after operation, and EB/TTC double staining was detected at 3 days after operation. A. Representative digital images of heart sections by Evans blue and TTC double staining, and the percentage of infarct area. The blue-stained portion indicates the normal region, the red-stained portion indicates the ischemic region, and the white portion indicates the infarcted region. The ratio of the infarct area of the largest heart section to the total area of the section was chosen to be the percentage of infarct area. B. Serum CK , CKMB levels, and serum LDH levels. Data are presented as mean ± SEM. (n = 6 in each group). * $p < 0.05$, ** $p < 0.01$, *** $p < 0.001$.

Supplementary Figure S2

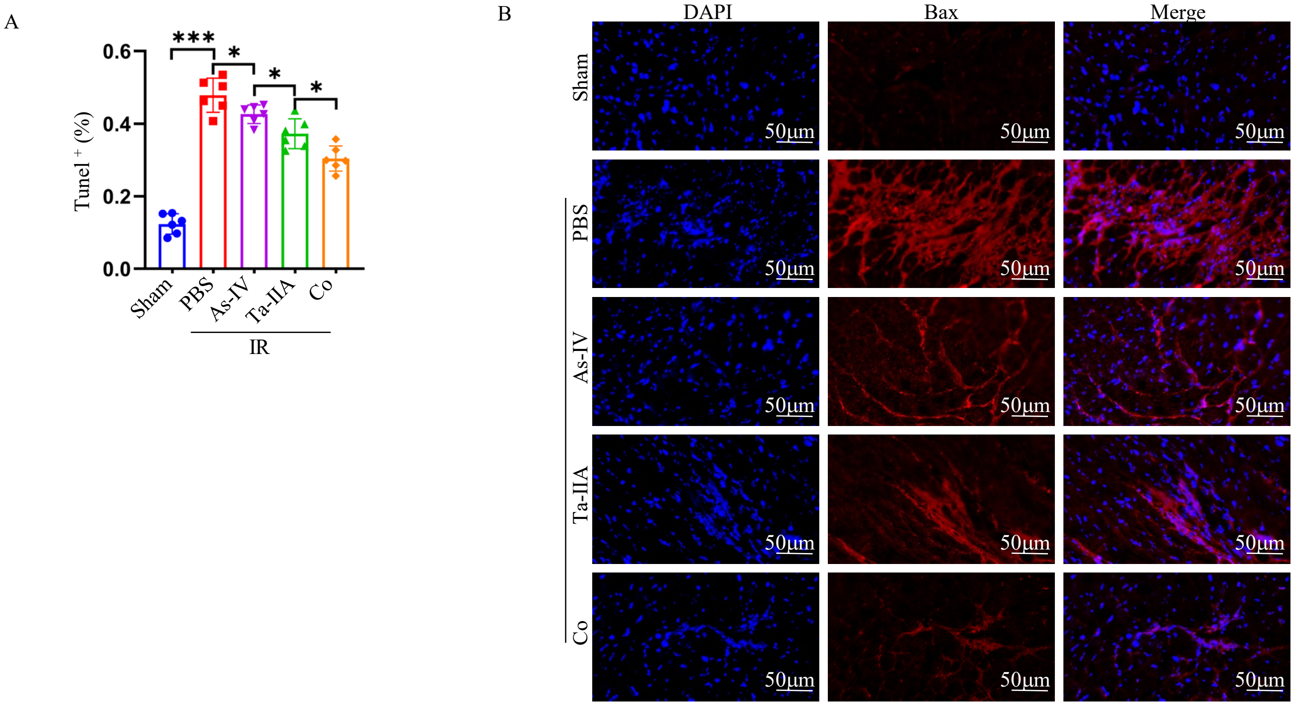

Supplementary Figure S2. A. The quantitative analysis of $Tunel^+$ cells of myocardial tissue in Figure2A. Data are presented as mean ± SEM. (n = 6 in each group). * $p < 0.05$, ** $p < 0.01$, *** $p < 0.001$. B. Representative immunofluorescence staining of Bax protein expression in cardiomyocytes of MIRI treated by Ta-IIA, As-IV, and Co. The Bax proteins were stained red, and the nuclei were detected by DAPI (blue).

Supplementary Figure S3

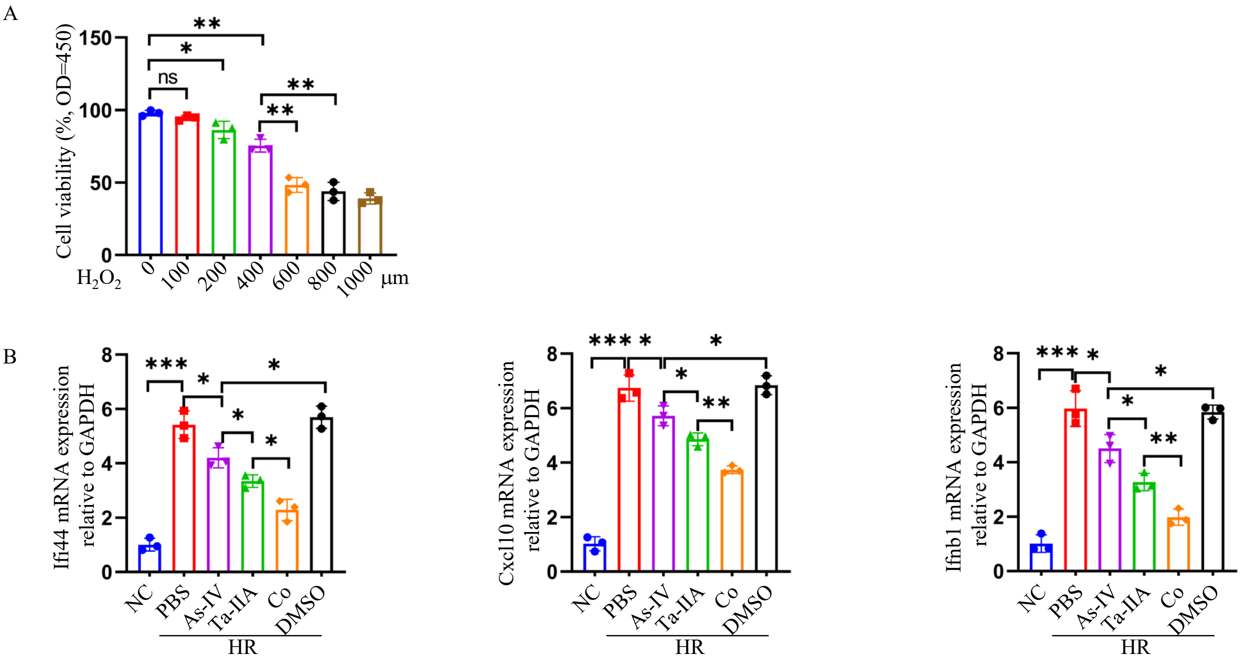

Supplementary Figure S3. A. Cell activity of HL1 cells incubated with different concentrations of H_2O_2 for 6 h. B. Quantification of mRNA expression of the cytokines Ifi44, Cxcl10 and Ifnb1 downstream of the STING pathway in HL1 cells induced by HR. Data are presented as mean ± SEM. (n = 3 in each group). * $p < 0.05$, ** $p < 0.01$, *** $p < 0.001$, ns = not statistically significant.

Supplementary Figure S4

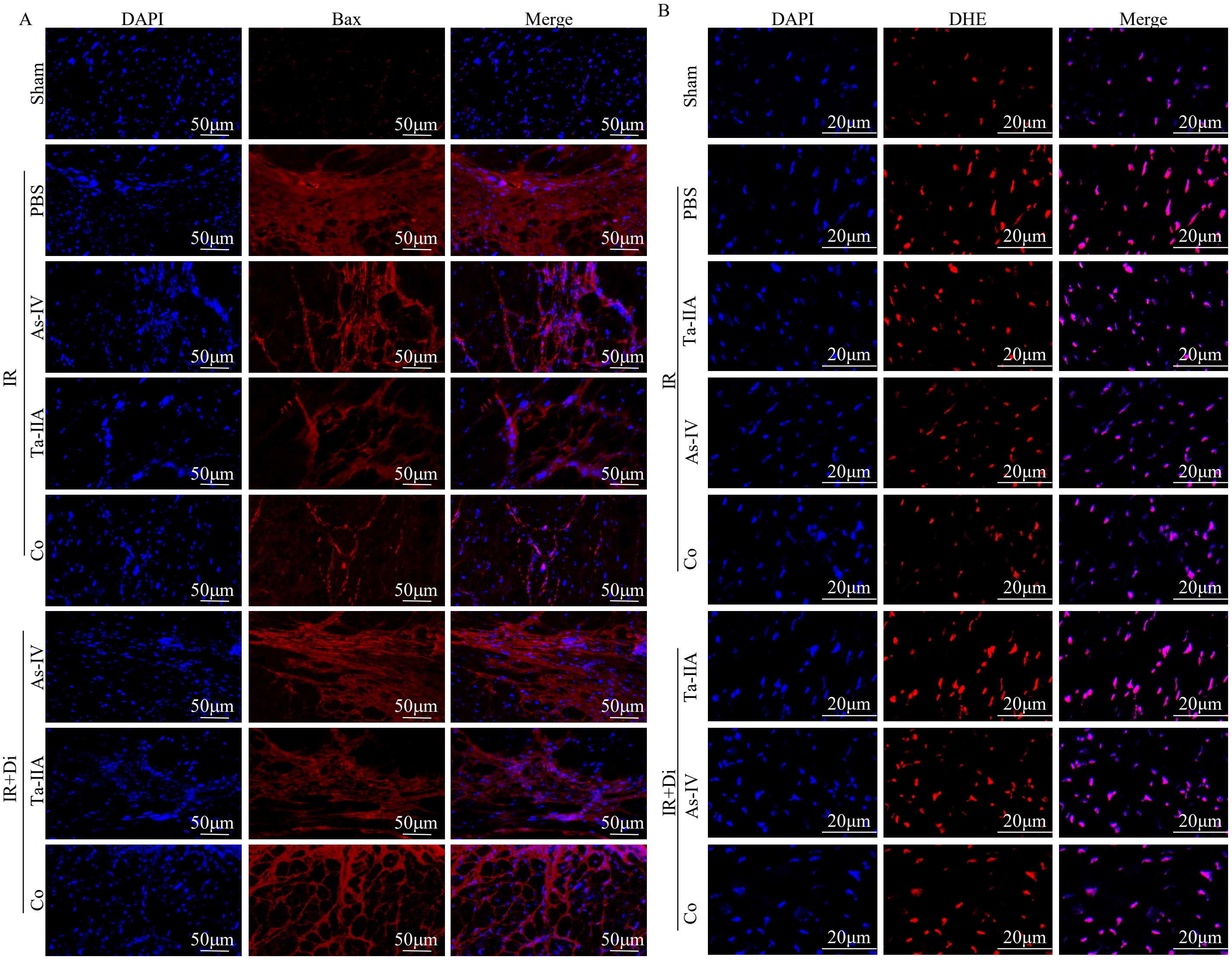

Supplementary Figure S4. A. Representative immunofluorescence staining of Bax protein expression in MIRI cardiomyocytes treated with Ta-IIA, As-IV and Co after agonist addition. The Bax proteins were stained red, and the nuclei were detected by DAPI (blue). B. Representative images of ROS content in cardiomyocytes. ROS was detected by DHE fluorescence staining (red), and nuclei were detected by DAPI (blue). scale bar = 20 μm.

Supplementary Figure S5

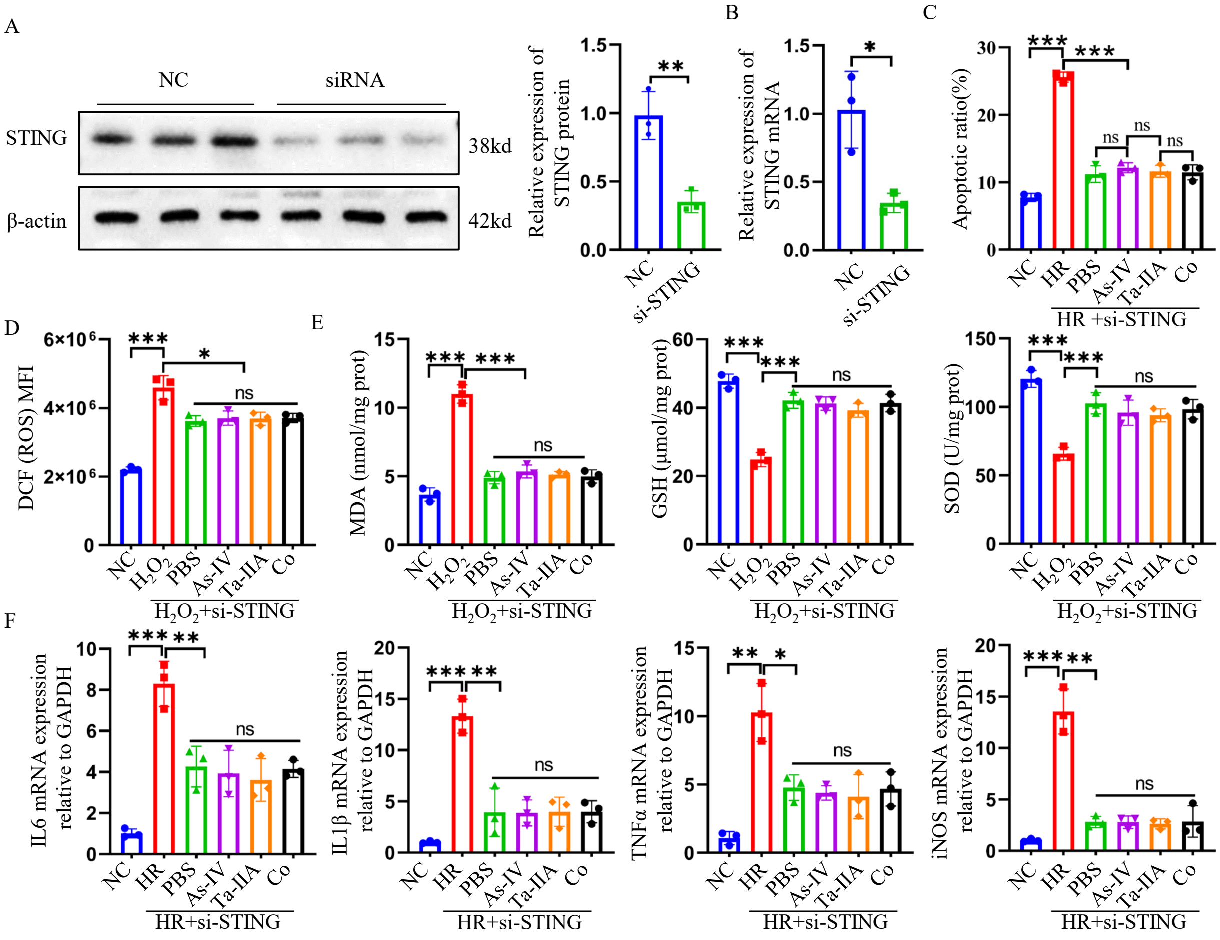

Supplementary Figure S5. The STING signaling pathway plays a key role in the anti-apoptotic, antioxidant and anti-inflammatory effects of Ta-IIA, As-IV and Co on HL1 cells of HR. A. The efficiency of STING siRNA was confirmed by western blot. B. The efficiency of STING siRNA was confirmed by qPCR. C. Quantitative analysis of the apoptosis rate of HL1 cells after STING siRNA. D. Quantitative analysis of the DCF mean fluorescence intensity of HL1 cells after STING siRNA. E. Cellular malondialdehyde(MDA) content, glutathione (GSH) activity, superoxide dismutase (SOD) activity of HL1 cells after STING siRNA. F. Quantitative analysis of cytokines IL-6, IL-1β, TNFα and iNOS mRNA in STING siRNA HL1 cells. Data are presented as mean ± SEM. (n = 3 in each group). * $p < 0.05$, ** $p < 0.01$, *** $p < 0.001$, ns = not statistically significant.

Supplementary Figure S6

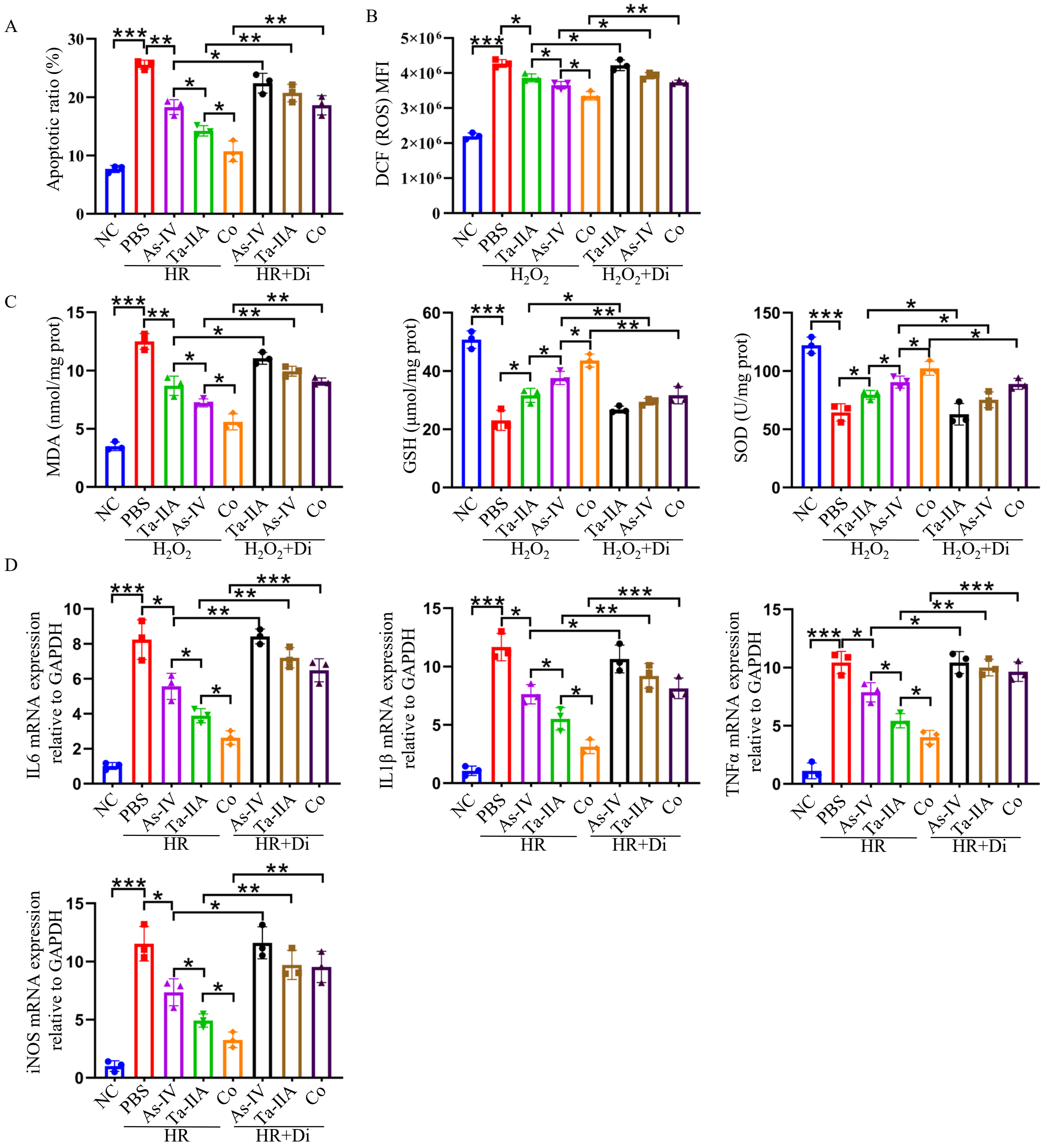

Supplementary Figure S6. The STING signaling pathway plays a key role in the anti-apoptotic, antioxidant and anti-inflammatory effects of Ta-IIA, As-IV and Co on HL1 cells of HR. A. Quantitative analysis of the apoptosis rate of HL1 cells after diabZI administration. B. Quantitative analysis of the DCF mean fluorescence intensity of HL1 cells after diabZI administration. C. Cellular MDA content, GSH activity, SOD activity of HL1 cells after diabZI administration. D. Quantitative analysis of cytokines IL-6, IL-1β, TNFα and iNOS mRNA in HL1 cells diabZI administration. Data are presented as mean ± SEM. (n = 3 in each group). * $p < 0.05$, ** $p < 0.01$, ***$p < 0.001$.
