# Supplementary material for: The combination of Tanshinone IIA and Astragaloside IV attenuates myocardial ischemia–reperfusion injury by inhibiting the STING pathway
Source: Chin Med. 2024 Feb 28;19:34. doi: 10.1186/s13020-024-00908-y (PMC10900662; doi:10.1186/s13020-024-00908-y)
Supplement: Supplementary file 2 — Additional file 2: Table S1. List of key chemicals. Table S2. List of commercial assays. Table S3. List of antibodies. Table S4. Primers used in qPCR assay. [file 13020_2024_908_MOESM2_ESM.docx]

Supplementary Table 1. List of Key Chemicals.

| **Chemicals** | **SOURCE** | **Cat No.** |
| --- | --- | --- |
| Tanshinone ⅡA | Chengdu Herbpurify | 568-72-9 |
| Astragaloside Ⅳ | Chengdu Herbpurify | 84687-43-4 |
| diABZI compound 3 | Selleck chem | 2138498-18-5 |
| Opti-MEM | Thermo Scientific | 31985088 |
| Lipofectamine 3000 | Thermo Scientific | L3000008 |
| Evans blue | Sigma-Aldrich | 314-13-6 |
| TTC | Solarbio | G3005 |
| RIPA protein lysis buffer | Beyotime | P0013B |
| PMSF | Servicebio | G2007-1 ML |
| Tween-20 | Sigma-Aldrich | P9416 |
| DMEM | Servicebio | G4511-500ML |
| TRIzol | Beyotime | R0016 |
| ECL | Beyotime | P0018S |

Supplementary Table 2. List of commercial assays.

| **Chemicals** | **SOURCE** | **Cat No.** |
| --- | --- | --- |
| BCA assay | Beyotime | P0010 |
| AnnexinV-APC/PI apoptosis detection Kit | Multisciences | AP107 |
| CK | Nanjing Jiancheng Bioengineering Institute | A032-1-1 |
| CKMB | Nanjing Jiancheng Bioengineering Institute | E006-1-1 |
| LDH Cytotoxicity Assay Kit | Nanjing Jiancheng Bioengineering Institute | A020-2 |
| TUNEL | Elabscience | E-CK-A321 |
| Dihydroethidium | Beyotime | S0063 |
| ROS | Beyotime | S0033S |
| MDA | Nanjing Jiancheng Bioengineering Institute | A003-1 |
| GSH | Nanjing Jiancheng Bioengineering Institute | A006-2-1 |
| SOD | Nanjing Jiancheng Bioengineering Institute | A001-3-1 |
| SynScript^®^ⅢRT SuperMix for qPCR (+gDNA Remover) kit | TSINGKE | TSK314S |
| 2×TSINGKE^®^Master qPCR Mix (SYBR Green I) kit | TSINGKE | TSE201 |

Supplementary Table 3. List of Antibodies.

| **ANTIBODY** | **SOURCE** | **Cat NO.** |
| --- | --- | --- |
| anti-Bax, rabbit monoclonal | Abmart | Tu333334s |
| anti-Bcl2, rabbit monoclonal | Abmart | TU323153s |
| anti-Caspase3,mouse monoclonal | Abmart | m005851 |
| anti-cGAS, rabbit polyclonal | Abclonal | A8335 |
| anti-Phospho-STING (Ser365) (D8F4W), rabbit monoclonal | Cell Signaling Technology | 72971S |
| anti-Phospho-IRF-3 (Ser396) (4D4G), rabbit monoclonal | Cell Signaling Technology | 4947 |
| anti-Phospho-TBK1/NAK (Ser172) (D52C2) | Cell Signaling Technology | 5483S |
| anti- STING, rabbit polyclonal | Proteintech | 19851-1-AP |
| anti- IRF3, rabbit polyclonal | Proteintech | 11312-1-AP |
| anti- TBK1, rabbit polyclonal | Proteintech | 28397-1-AP |
| anti-α-actin, rabbit monoclonal | Proteintech | 23660-1-AP |
| HRP Goat Anti-Rabbit IgG | Abclonal | AS014 |
| HRP Goat Anti- Mouse IgG | Abclonal | AS003 |

Supplementary Table 4. Primers used in qPCR Assay.

| **Primers**  **(mouse)** | **Forward (5′-3′)** | **Reverse (5′-3′)** | |
| --- | --- | --- | --- |
| IL6 | ACTTCCATCCAGTTGCCTTCTTGG | TTAAGCCTCCGACTTGTGAAGTGG |  |
| IL1β | GCACAGTTCCCCAACTGGTA | TGTCCCGACCATTGCTGTTT |  |
| TNFα | GGTGCCTATGTCTCAGCCTCTT | GCCATAGAACTGATGAGAGGGAG |  |
| iNOS | GAGACAGGGAAGTCTGAAGCAC | CCAGCAGTAGTTGCTCCTCTTC |  |
| Ifnb1 | CAGCTCCAAGAAAGGACGAAC | GGCAGTGTAACTCTTCTGCAT |  |
| Ifi44 | AACTGACTGCTCGCAATAATGT | GTAACACAGCAATGCCTCTTGT |  |
| cxcl10 | CCAAGTGCTGCCGTCATTTTC | GGCTCGCAGGGATGATTTCAA |  |
| STING siRNA | CCACUGUAUGGCUAUGAUUTT | AAUCAUAGCCAUACAGUGGTT |  |
| GAPDH | GCTTCTAGGCGGACTGTT AC | CCATGCCAATGTTGTCTCTT |  |
| β-actin | GGCTGTATTCCCCTCCATCG | CCAGTTGGTAACAATGCCATGT |  |
